# From forest floor to doctor’s office: the immunological journey of *Borrelia burgdorferi* through vertebrate hosts

**DOI:** 10.3389/fimmu.2026.1811554

**Published:** 2026-03-31

**Authors:** Preeti Singh, Troy Bankhead

**Affiliations:** Department of Veterinary Microbiology and Pathology, Washington State University, Pullman, WA, United States

**Keywords:** lyme disease, *Borrelia burgdorferi*, spirochete, host-pathogen interactions, persistence, tissue colonization, immune evasion, antigenic variation

## Abstract

Lyme disease, caused by the spirochete *Borrelia burgdorferi*, is the most prevalent vector-borne infection in the Northern Hemisphere and continues to expand geographically. Although *B. burgdorferi* has a streamlined genome and minimal virulence repertoire, it establishes persistent infection through coordinated modulation of mammalian host immune responses. Here, we synthesize recent advances in the immunobiology of *B. burgdorferi* using a stage-structured framework that traces a tick-mediated vertebrate infection through systemic dissemination, tissue colonization, and chronic immune engagement. Emphasis is placed on post-2020 insights enabled by intravital imaging, single-cell transcriptomics, spatial profiling, and systems immunology, which have refined our understanding of endothelial transmigration, tissue-specific immune conditioning, disruption of germinal center responses, and failure of sterilizing immunity. We highlight how antigenic variation at the *vls* locus, complement evasion, and coordinated adhesin networks support dissemination and long-term tissue residency, while adaptive immune responses are redirected toward extrafollicular, non-sterilizing trajectories. These immune strategies differentially shape infection outcomes across host species, supporting asymptomatic persistence in reservoir hosts while driving inflammatory disease in humans. The review further examines antigen persistence, immune stalemate, and post-treatment inflammatory sequelae, integrating translational advances in diagnostics and prevention. By integrating an ecological context with mechanistic immunology and clinical insight, this review presents a contemporary framework for understanding how immune modulation by *B. burgdorferi* across spatial and temporal scales shapes host-pathogen coevolution and informs improved diagnostic strategies, vaccine development, and therapeutic intervention.

## 1 Introduction

From the shaded canopy of deciduous forests, where *Ixodes* ticks thrive amid decomposing leaf litter to the molecular battlegrounds of mammalian tissue, a journey unfolds that traverses not only ecosystems but the intricate terrain of the vertebrate immune system. *Borrelia burgdorferi* (also called *Borreliella*), the etiologic agent of Lyme disease, occupies a remarkable position at the intersection of ecology, vector biology, and host immunity (1–3). Despite its reduced genome, limited metabolic capacity, and absence of classical virulence factors, *B. burgdorferi* disseminates across diverse vertebrate hosts (4–6). The success of this minimalist pathogen hinges not on brute virulence, but on its ability to navigate and modulate host immune responses in ways that enable long-term persistence (7). Since the initial recognition of Lyme disease nearly five decades ago (8), substantial progress has been made in elucidating the complex biology and pathogenesis of this spirochete. Nevertheless, many fundamental questions remain unresolved. This review commemorates the 50-year milestone by synthesizing recent advances in our understanding of the immunological trajectory of the spirochete through its vertebrate hosts.

Framing *B. burgdorferi* infection as a biological odyssey, this review is organized into thematic stages corresponding to major immune checkpoints encountered by the spirochete during vertebrate infection. Foundational concepts are illustrated schematically in the accompanying figures, allowing the main text to emphasize recent insights emerging from the post-pandemic era. Host immune encounters are influenced well before infection occurs, as tick phenology and reservoir host diversity shape exposure risk and define the initial immune landscape (9, 10). The ensuing host-pathogen interaction unfolds from skin to vasculature and tissue niches, progressing from early innate activation toward adaptive reshaping and tolerance-like states. We therefore begin on the forest floor, where ecological forces governing vector-host interactions set the stage for transmission of the pathogen. Successful colonization of the tick midgut and subsequent transmission into vertebrate skin mark a critical immunological threshold for the spirochete (11). The dermal tick feeding site becomes the first immune arena, where the tick’s salivary immunomodulators suppress host defenses and permit early spirochete establishment. Subsequent sections examine how coordinated programs of motility, adhesion, endothelial engagement, and immune interference enable systemic dissemination and tissue niche occupation. Particular emphasis is placed on the pathogen’s remarkable capacity to evade and recalibrate both innate and adaptive immune responses through complement evasion, antigenic variation, and phenotypic plasticity, including proposed biofilm-like or intracellular persistence (12–14). This framework highlights the contrasting immunological landscapes encountered in reservoir versus incidental hosts, illuminating how long-term persistence is tolerated in the former yet often manifests as inflammatory pathology in the latter (15). The journey culminates in the doctor’s office, where delayed diagnosis, therapeutic challenges, and post-treatment Lyme disease syndrome reflect the downstream consequences of prolonged immune-pathogen interaction and immune remodeling. A recurring theme throughout is the failure of sterilizing immunity and durable immune memory, with important implications for diagnostics, reinfection, and post-treatment outcomes (16).

Collectively, this review reframes the pathogenesis of *B. burgdorferi* not as a linear infectious process, but as a dynamic lifecycle shaped by immune pressures across ecological and anatomical contexts. Ecology, vector biology, and clinical medicine emerge as interconnected forces that govern host recognition, pathogen evasion, and disease outcome. This synthesis underscores how sustained interactions between the spirochete and its vertebrate hosts have shaped finely tuned immune modulation strategies. Integrating host immune dynamics across spatial and temporal scales provides critical insights into host-pathogen coevolution, informs immunologically grounded intervention strategies, and offers a framework for anticipating Lyme disease emergence under changing environmental conditions.

## 2 The odyssey begins: forest floor

Lyme disease (LD), first described in the mid-1970s in Lyme, Connecticut (8), has since emerged as the most prevalent vector-borne illness in the Northern Hemisphere, with reported cases continuing to rise in both magnitude and geographic distribution (17). Although surveillance data from the U.S. Centers for Disease Control and Prevention documented over 89,000 reported cases in 2023 (Lyme Disease Surveillance and Data: https://www.cdc.gov/lyme/data-research/facts-stats/index.html/ Accessed 10 February 2026), true numbers are estimated to be 8 to 10 times higher, underscoring the scale of ongoing transmission (John Hopkins LD impact report, 2024: https://www.hopkinslyme.org/research/identifying-the-geographic-leading-edge-of-lyme-disease-using-google-health-trends/). To effectively disrupt the transmission cycle of *B. burgdorferi* and reduce human infection risk, it is essential to understand the ecological context in which the pathogen is maintained, establishing the immunological conditions that enable long-term persistence.

The forest floor represents an upstream ecological checkpoint that preconfigures the immunological context of subsequent infection, the ecological interface where interactions among reservoir hosts, vectors, and pathogen shape transmission dynamics. Within temperate forest ecosystems, the spirochete is maintained in a highly specialized enzootic cycle involving *Ixodes* spp. ticks as obligate hematophagous vectors and small mammals as primary reservoirs (7). Within this ecological niche, the spirochete resides in a state of immune equilibrium with competent reservoir hosts such as *Peromyscus leucopus*, characterized by persistent, asymptomatic colonization, reflecting a state of immune accommodation rather than sterilizing immunity (6, 7). This reservoir-adapted equilibrium represents the foundational immune state from which downstream transmission emerges. The transmission cycle continues when uninfected larval or nymphal ticks acquire the spirochete while taking a bloodmeal from these hosts. Once ingested, the spirochetes colonize the tick midgut, a critical anatomical niche that supports its survival through molting stages and primes it for transmission during subsequent feeds (18). At this stage incidental hosts, including humans, become infected but do not contribute to onward transmission (6, 7). Host competence varies widely across vertebrate species and plays a critical role in shaping local enzootic dynamics. While small mammals and some ground-foraging birds efficiently transmit spirochetes to feeding ticks, larger vertebrates such as white-tailed deer are reservoir-incompetent but act as amplification hosts by sustaining tick populations required for vector reproduction (6). Additionally, environmental perturbations including climate change, habitat modification, and shifts in host distributions are accelerating the geographic expansion of both *Ixodes* vectors and *B. burgdorferi* (19–23). Beyond altering exposure risk, these forces shape the immune landscapes encountered by the pathogen in incidental hosts by modifying timing of transmission, host composition, and vector density. Together, these upstream ecological processes preconfigure the infection context, setting the stage for subsequent dissemination, persistence, and disease. These reservoir-adapted immune equilibria form the backdrop against which tick-borne entry into the vertebrate host unfolds.

## 3 Departure gate: tick-borne entry into the mammalian host

Transmission begins when an infected *Ixodes* tick deposits *B. burgdorferi* into the dermis during blood feeding, initiating the earliest host-pathogen interactions that shape the subsequent infection dynamics. At the bite site, vector-derived salivary proteins modulate complement activation, neutrophil recruitment, and local cytokine signaling, creating a transient immunological environment that facilitates spirochete survival and establishment. The tick bite site therefore functions as a biological “border crossing”, representing the first immunological checkpoint of mammalian infection as spirochetes transition from the arthropod vector into a fundamentally different immune landscape and establish their initial foothold in host tissue.

### 3.1 Saliva-assisted transmission and dynamic tick salivary remodeling

**Figure 1:**
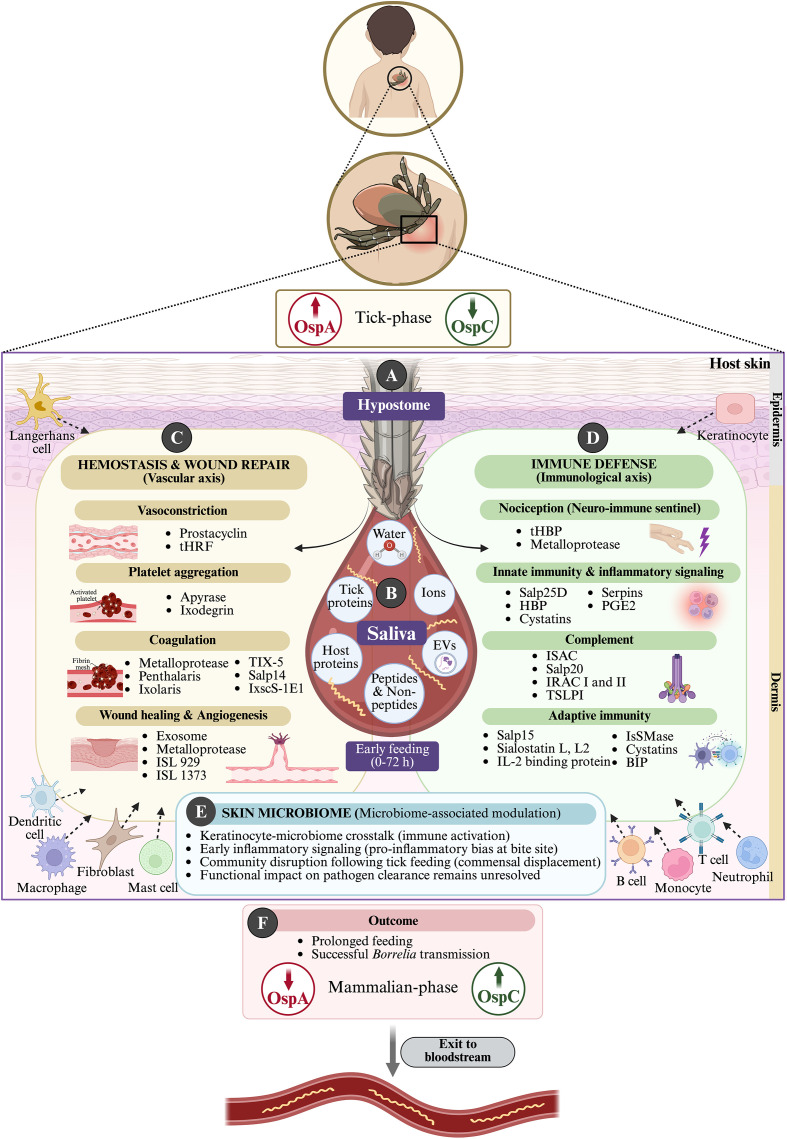
Departure gate: tick-borne entry of *B. burgdorferi* into the mammalian host. (Top) *Ixodes* tick attachment establishes a localized feeding lesion at the skin surface, marking the departure gate for *Borrelia burgdorferi* transmission from vector to the vertebrate host. During feeding, spirochetes transition from a tick-adapted (OspA↑, OspC↓) to a mammal-adapted state (OspA↓, OspC↑). **(A, B)** Prolonged blood feeding by *Ixodes* ticks is enabled by penetration of the hypostome through the epidermis and dermis, forming a cemented feeding lesion and sustaining continuous saliva delivery. Tick saliva, enriched in water, ions, peptides and non-peptides molecules, proteins of both tick and host origin, and extracellular vesicles, creates a localized, saliva-conditioned cutaneous microenvironment. **(C)** Hemostasis and wound repair (vascular axis). Tick salivary effectors disrupt hemostasis and wound repair that normally limit blood loss and restore tissue integrity. They target vasoconstriction, platelet aggregation, coagulation, wound healing, and angiogenesis. These functions are mediated by families of salivary molecules, including prostaglandins and nitric oxide carriers, Kunitz domain-containing proteins, metalloproteases, and exosome-associated factors. Together, these actions sustain blood flow and delay tissue restoration at the bite site. **(D)** Immune defense (immunological axis). In parallel, tick saliva suppresses early neuro-immune sensing and immune activation at the bite site. Nociception, innate immunity, complement activity, and adaptive priming are modulated by salivary protein families such as lipocalins, cystatins, serpins, evasins, Salp proteins, complement inhibitors, and CD4^+^ T cell-targeting immunomodulators. These effects impair leukocyte recruitment, dampened inflammatory amplification, and delayed immune recognition. Cytokine and chemokine signaling are altered, disrupting coordinated immune responses by keratinocytes, Langerhans cells, dendritic cells, macrophages, neutrophils, and mast cells within the epidermis and dermis. **(E)** The skin microbiome constitutes an additional modulatory layer at the bite site. It engages in keratinocyte-microbiome crosstalk and contributes to early pro-inflammatory signaling. Tick feeding can disrupt microbial community structure, although the functional consequences for pathogen clearance remain unresolved. **(F)** (Outcome) Vascular disruption, immune modulation, and microbiome perturbation together establish a transient immune-privileged niche. The environment supports prolonged tick feeding and facilitates successful *B. burgdorferi* transmission. Spirochetes subsequently exit the dermis and enter the bloodstream.

During tick feeding, spirochetes residing in the midgut become motile, migrate to the salivary glands, and are deposited into the dermal milieu together with tick saliva (**Figure 1**). As pool feeders requiring prolonged attachment, *Ixodes* ticks anchor firmly to host skin using a cement-like salivary matrix that secures the barbed hypostome and chelicerae within the epidermis and dermis while simultaneously deploying a complex arsenal of salivary molecules that suppress hemostasis, pain, itch, inflammation, and wound repair (6, 24). These coordinated activities create a permissive, immunologically altered bite-site microenvironment that enables sustained feeding and facilitates pathogen transmission, a process termed saliva-assisted transmission (SAT) (24, 25). Notably, the composition of tick saliva changes dynamically during feeding, with functionally related but antigenically distinct proteins secreted in successive ~24-hour intervals, a strategy that may parallel spirochetal antigenic variation in limiting host immune recognition (26). Refining the classical SAT model, recent proteomic studies demonstrate that *B. burgdorferi* itself reshapes tick salivary output, enriching factors that favor spirochete survival while reducing components predicted to generate reactive oxygen species (27).

### 3.2 Tick mediated immune modulation at the bite site

Over the past two decades, extensive work has interrogated the tick-host cutaneous interface in increasing mechanistic depth, a body of knowledge comprehensively synthesized in a recent review (24). At the bite site, early host responses include rapid recruitment of neutrophils and monocytes and activation of skin-resident immune populations, including dendritic cells, keratinocytes, dermal T cells, and Langerhans cells, with a concurrent bias toward Th2-skewed immunity at the evolving bite lesion (24). To counter these defenses, ticks deploy immunomodulatory salivary factors that dampen innate, complement, and adaptive immune pathways while promoting vasodilation (24). Human *ex-vivo* model systems have provided direct evidence that tick feeding profoundly reshapes the cutaneous immune landscape, interfering with effective primary immune responses (28). Recent work has identified specific salivary immunosuppressive protein (IpSAP), which inhibits of LTβR signaling, a pathway that contributes to host resistance against tick-borne Lyme spirochetes (29) and demonstrated that epidermal sentinels as Langerhans cells can be driven toward tolerance-like or regulatory trajectories during concurrent tick feeding and *B. burgdorferi* exposure. Together, these mechanisms establish a transient immune-privileged niche that supports early spirochete survival (30).

### 3.3 Coinfections as modifiers of the infectious trajectory

Ticks frequently transmit multiple microbes rather than a single pathogen during a feeding event, altering both host immune responses and disease severity (31). *Ixodes* ticks commonly harbor *Anaplasma phagocytophilum* and *Babesia microti*, and coinfections with *B. burgdorferi* are well documented in endemic regions (31–34). Within this infectious journey, these organisms function as “co-passengers”, shaping immune polarization, pathogen burden, and tissue-specific outcomes. Clinical studies indicate that approximately 40 percent of babesiosis cases and up to 13 percent of human granulocytic anaplasmosis show concurrent infection with *B. burgdorferi*, highlighting the ecological overlap among these pathogens (31, 33). Experimental studies suggest that *B. microti* coinfection can increase *Borrelia* burden in the brain and potentially influence the severity of neuroborreliosis (35). Coinfection with *A. phagocytophilum* has also been associated with enhanced endothelial inflammatory responses and increased production of cytokines and matrix metalloproteases that may facilitate vascular permeability and tissue invasion (36). More broadly, polymicrobial exposure can also reshape systemic immune polarization, with altered interferon responses reported in the host infected with multiple tick-borne pathogens (31). Together, these observations suggest that Lyme disease frequently unfolds within a polymicrobial ecological context in which immune responses are shaped not only by *B. burgdorferi* but by the broader community of tick-borne microbes.

### 3.4 Spirochete motility and early cutaneous dissemination

Successful entry of the spirochete into the host depends not only on immune evasion but also on effective navigation within the cutaneous environment. Chemotaxis has emerged as a key determinant of this process, with the methyl-accepting chemotaxis protein (MCP5) shown to be required for efficient transmission to the mammalian host (37). Consistent with this, transmission and early establishment imposes population bottlenecks such that only a subset of spirochetes successfully transitions from tick to host and persist long enough to seed systemic infection (38). Together, these factors establish a transient immune-privileged niche that enables *B. burgdorferi* to exit the dermis within approximately 72 hours post-tick attachment, marking the departure from the skin and initiating the next phase of the journey: systemic dissemination via vascular and lymphatic networks under continuous immune surveillance.

## 4 On the move: the vascular highway

After establishing a localized dermal foothold at the site of inoculation, often marked clinically by erythema migrans, *B. burgdorferi* embarks on a highly orchestrated journey along the vascular “highway”. Dissemination proceeds through coordinated expansion in the dermis, migration through the connective matrix, and stepwise engagement of vascular and lymphatic barriers. Completing this transition requires traversal of structural and immunological checkpoints formed not only by endothelial cells, but also shaped by pericytes, basement membrane, smooth muscle cells, fibroblasts, and resident immune sentinels. Decades of imaging studies, complemented by recent reductionist model systems, have revealed this process as a staged, multistep sequence of integrating motility, adhesion, endothelial modulation and immune evasion.

### 4.1 Dermal motility and navigation toward vascular entry

**Figure 2:**
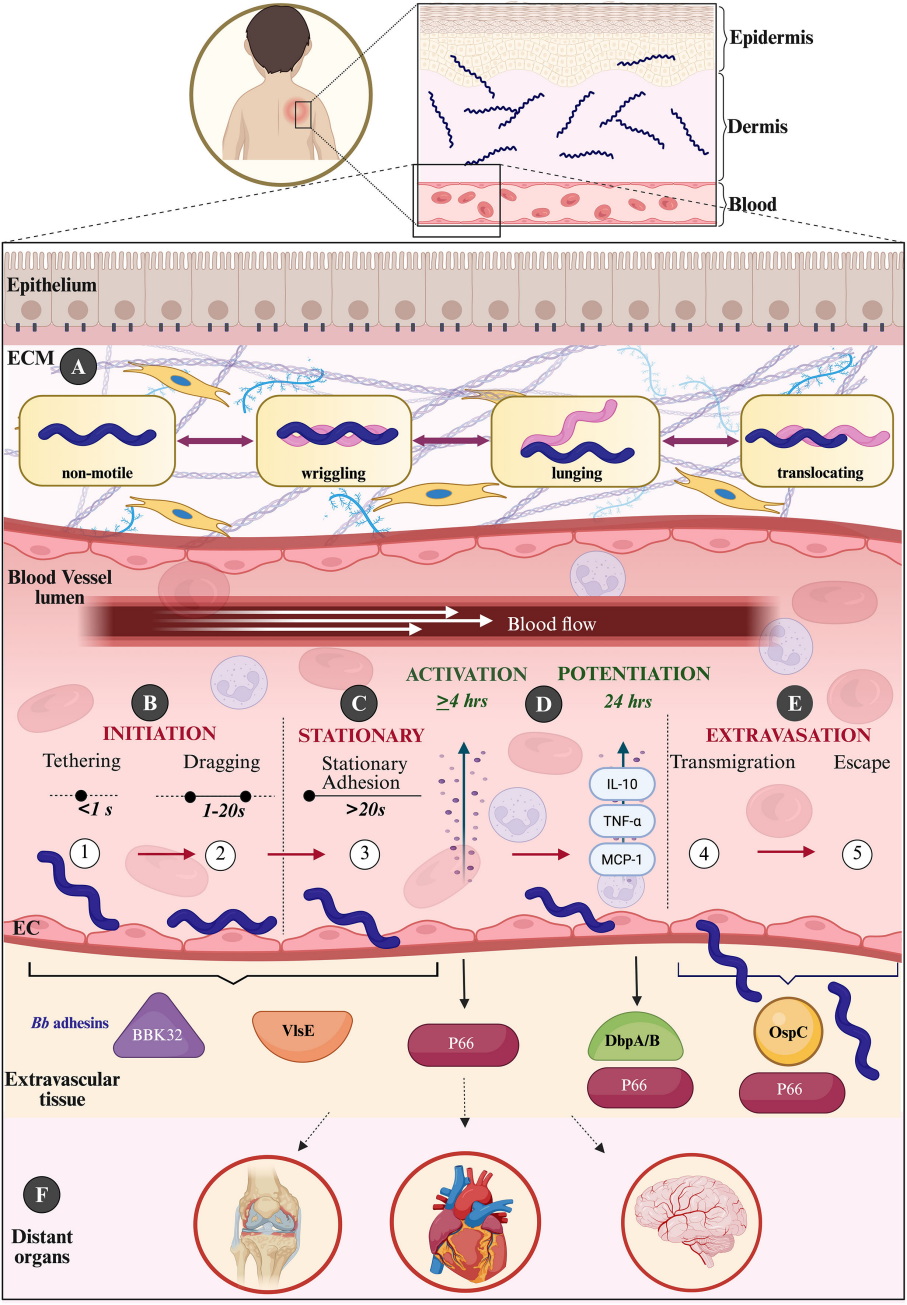
On the move: vascular highway– multistage dissemination of *B. burgdorferi* from dermis to distant tissues. **(A)** Dermal introduction and ECM motility. Following tick-mediated inoculation, *B. burgdorferi* establishes a localized dermal foothold. It migrates through the extracellular matrix (ECM) using distinct motility states– non-motile, wriggling, lunging, and translocating. These reflect mechanosensitive adhesion-detachment cycles that enable navigation of dense connective tissue. **(B)** Vascular encounter and initiation under shear. Upon reaching the microvasculature, spirochetes undergo transient tethering (<1 s) and dragging (1–20 s) under blood flow. These interactions are mediated primarily by BBK32 and VlsE. BBK32 acts as molecular “velcro,” binding fibronectin to initiate tethering and glycosaminoglycans to stabilize dragging. Plasma fibronectin is recruited to form load-bearing adhesion complexes, arresting spirochetes under flow. **(C)** Stationary adhesion at endothelial junctions. Prolonged stationary adhesion (>20 s) occurs predominantly at endothelial cell-cell junctions. This positions spirochetes for subsequent vascular escape. **(D)** Endothelial activation and potentiation. Endothelial engagement triggers an activation phase (≥4 h), characterized by cytokine release and increased permeability. A subsequent potentiation phase (~24 h) renders the endothelium permissive to transmigration. Recruited neutrophils contribute inflammatory mediators, including TNF-α, MCP-1, and IL-10, which reinforce endothelial activation. **(E)** Transcellular transmigration and extravasation. Spirochetes predominantly traverse the endothelium via a transcellular route. This process requires the integrin-binding porin P66 and temporally coordinated adhesins. DbpA/B contribute during potentiation, while OspC functions at the extravasation step, enabling escape into extravascular tissue. **(F)** Dissemination to distant organs. Following vascular exit, *B. burgdorferi* spreads to peripheral tissues, including joints, heart, and nervous system. Together, the schematic depicts dissemination as an orchestrated, multistep process integrating biomechanical motility, sequential adhesin engagement, endothelial modulation, and immune co-option. The vasculature thus acts as an active interface, where pathogen tricks the host into negotiating the terms of permissive movement. (EC, Endothelial cells).

Early studies established that *B. burgdorferi* moves through the collagen-rich dermis using distinct motility states, such as translocating, lunging, wriggling, and non-motile, that are governed by transient ECM adhesion and matrix density (39) (**Figure 2**). These adhesion-detachment cycles reveal motility as a mechanosensitive behavior rather than merely flagellar-driven propulsion. Remarkably, *B. burgdorferi* can penetrate pores smaller than its own diameter, emphasizing its exceptional deformability and adaptive biomechanics (39). Three-dimensional tissue-engineered models demonstrate nondirectional migration toward dermal microvessels via trial-and-error exploration of endothelial junctions (40). Iterative reversals allow navigation around collagen fibers and steric obstacles, enabling advancement toward vascular entry while minimizing tissue disruption and immune activation (40). Recent volumetric imaging reframed early vascular engagement as a deliberate, spatially guided process (41). As the spirochete approaches the vasculature, it aligns along pericyte-enriched microdomains characterized by thin basement membranes and fibronectin-rich interfaces that serve as anatomical gateways for stepwise transcellular intravasation (41). Parallel studies reveal lymphatic entry through both transcellular and paracellular routes, facilitated by the structurally discontinuous lymphatic architecture, establishing lymphatics as a preferred route for early systemic spread (41). Limited shedding of outer membrane vesicles and tick-derived proteins early in infection reflects a dual strategy of mechanical opportunism and molecular restraint, allowing quiet vascular entry of *B. burgdorferi* while evading immune detection (41).

### 4.2 Adhesion-mediated vascular capture under shear stress

Once in circulation, *B. burgdorferi* encounters vascular shear forces that necessitate a finely tuned adhesin network. Intravital microscopy and bioluminescent imaging enabled the real-time visualization of spirochete-endothelial interactions in mice, identifying three sequential stages of vascular interactions: short-term tethering (<1 s over 100 µm), dragging (1–20 s), and stationary adhesions (>20 s), preceding extravasation (42). These steps are mediated by coordinated engagement of host fibronectin and glycosaminoglycans, with BBK32 initiates transient tethering and stabilizing dragging. VlsE, traditionally known for antigenic variation, binds dermatan sulfate to facilitate efficient vascular capture (43, 44). Together, VlsE and BBK32 account for most transient microvascular tethering events observed *in vivo* (45). *B. burgdorferi* BB0406 form additional interactions with laminin (46) and polymerized fibronectin sheaths generate load-bearing adhesion complexes stabilized via catch-bonds (47). Together, these mechanisms enable spirochetes to resist shear while positioning for endothelial exit.

### 4.3 Endothelial activation and transcellular extravasation

Stable adhesion alone is insufficient for vascular escape. Following BBK32- and VlsE- mediated engagement, endothelial cells undergo activation marked by cytokine release and transient increases in permeability reinforced by recruited neutrophils (48). A subsequent 24-hour potentiation phase renders the endothelium permissive to transmigration, during which the integrin-binding porin P66 becomes essential for transcellular passage (49). Additional adhesins, including OspC, DbpA/B, and RevA/B act at distinct kinetic stages, with OspC at extravasation and DbpA/B during potentiation, forming a coordinated adhesion network that secures and stabilizes footholds required for successful extravasation (45, 50, 51). Complementary *in vitro* studies demonstrated that live spirochetes induce a transient surge in endothelial traction forces followed by a prolonged reduction in motility, accompanied by early activation of innate immune pathways, including NF-κB and TNF-α signaling (52). These findings highlight immune-mechanical crosstalk through which *B. burgdorferi* transiently perturbs endothelial biomechanics to facilitate dissemination while restoring vascular homeostasis to evade prolonged inflammation. High-resolution assays reveal that *B. burgdorferi* predominantly uses a transcellular pathway, entering, traversing, and exiting individual endothelial cells rather than slipping between them (53). Pharmacologic studies identified host small GTPases as critical mediators: Cdc42 is required for internalization, while Rac1 mediates cytoplasm egress (53). The process exemplifies the spirochete’s ability to exploit host mechanics and immune signaling to engineer its own passage. Vascular permissiveness varies by endothelial identity. Spirochetes readily adhere to and extravasate through non-specialized endothelium, whereas brain microvasculature remains largely restrictive even under inflammatory conditions (54), consistent with spirochetes localization to the dura mater rather than brain parenchyma (55, 56). These differences highlight how endothelial phenotype and local shear environments shape the geography of dissemination.

Collectively, these studies converge on a model in which dissemination is not a passive breach of the vasculature but a regulated, bidirectional dialogue between pathogen and endothelium. The spirochete’s journey from skin to deep tissue depends on biomechanical versatility to traverse dense matrices, a coordinated adhesin network to mediate sequential vascular interactions, and engagement of immune and mechanical signaling pathways that reshape endothelial behavior. In this view, the vascular highway is not merely a conduit for spread but an active stage where the pathogen tricks the host into negotiating a permissive environment for dissemination.

## 5 Scenic stops: tissue colonization and niche occupation

**Figure 3:**
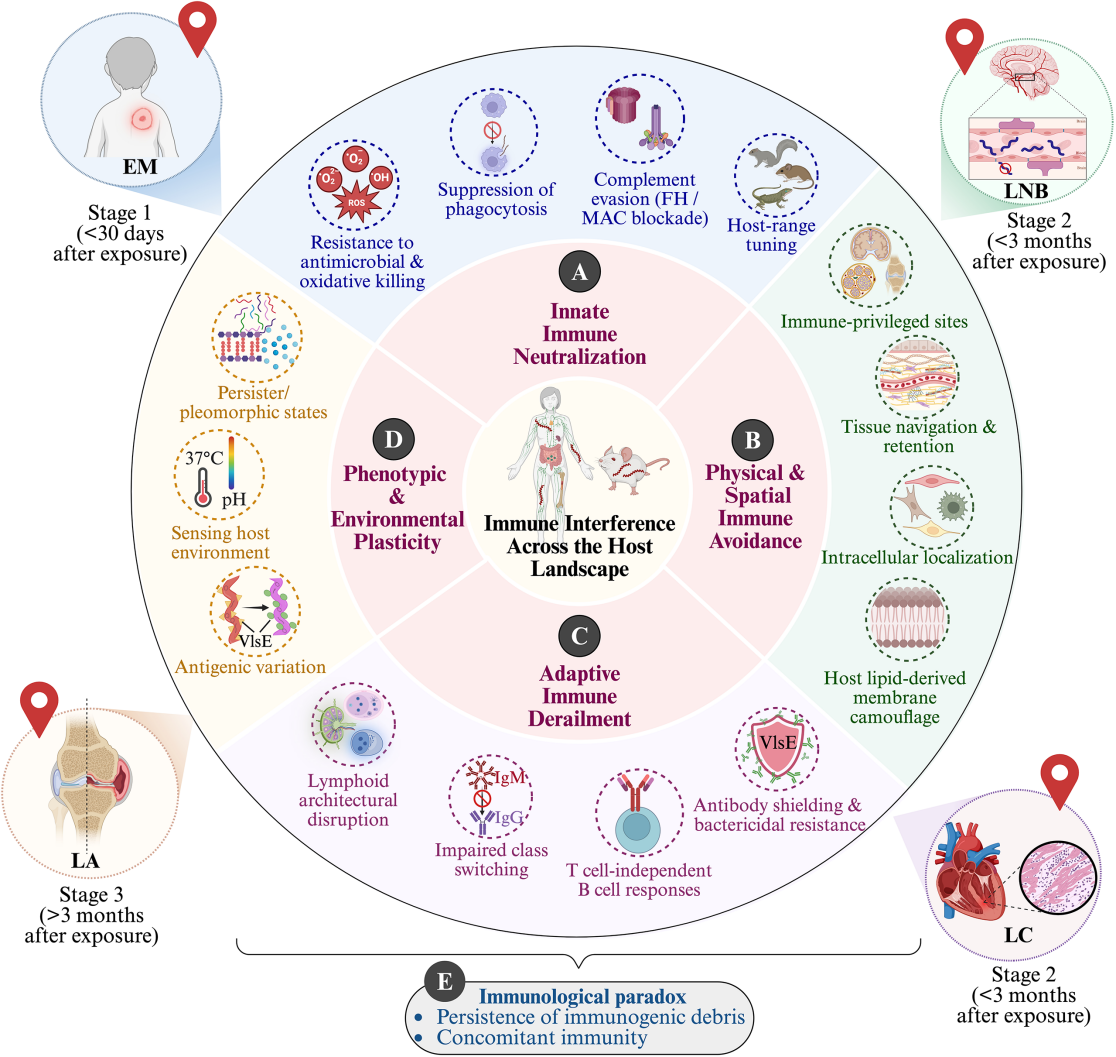
Scenic stops and extended stay: immune interference across the host landscape shapes tissue colonization, persistence, and clinical manifestation during *B. burgdorferi* infection. This schematic integrates pathogen-intrinsic strategies and host immune disruptions across spatial, temporal, and immunological scales. It frames the journey from early tissue colonization to prolonged immune stalemate. The central panel depicts the host landscape, emphasizing the translational continuum between experimental host models and human disease. Surrounding concentric rings organize immune interference by function rather than by individual molecules. The inner pink ring delineates four major modes of host-pathogen interaction that shape immune interference and persistence. The outer ring depicts representative mechanisms corresponding to each immune-interference category, illustrated as discrete modules. **(A)** Innate immune neutralization encompasses early strategies that limit host antimicrobial containment. These include resistance to antimicrobial and oxidative killing, suppression of phagocytosis, complement evasion via Factor H recruitment and membrane attack complex (MAC) blockade, and host-range tuning. **(B)** Physical and spatial immune avoidance includes mechanisms that reduce immune access through tissue-level positioning. They include occupation of immune-privileged sites, navigation and retention within extracellular matrix, intracellular localization, and acquisition of host-derived lipids that camouflage the bacterial membrane. **(C)** Adaptive immune derailment represents disruption of effective humoral immunity during prolonged infection. Strategies include antibody shielding, resistance to bactericidal antibodies, preferential induction of T cell-independent B cell responses, impaired immunoglobulin class switching, and lymphoid architectural disruption. **(D)** Phenotypic and environmental plasticity highlights pathogen-intrinsic, environmentally responsive state changes that enable persistence across heterogeneous host niches. These include antigenic variation mediated by recombination at *vls* locus, sensing of host environmental cues, and formation of persister or pleomorphic states. Together, these processes progressively decouple immune activation from effective clearance culminating in an **(E)** immunological paradox, shown at the bottom of the schematic, in which immunogenic debris persists, and concomitant immunity develops without sterilizing resolution. Clinical manifestations are positioned as peripheral landmarks (“scenic stops”) along the host journey, reflecting tissue-specific visibility of underlying immune-pathogen interactions. Erythema migrans (EM) represents early cutaneous colonization (Stage 1). Lyme neuroborreliosis (LNB) and Lyme carditis (LC) reflect dissemination into immune-privileged or systemic tissues (Stage 2). Lyme arthritis (LA) marks a later “scenic stop” associated with prolonged immune engagement and adaptive immune dysfunction (Stage 3). Placement of these manifestations emphasizes that clinical phenotypes emerge from the combined effects of spatial localization, phenotypic plasticity, and progressive immune derailment rather than from any single evasion mechanism. Overall, this figure illustrates how *B. burgdorferi* persistence arises from coordinated interference with innate and adaptive immunity across tissues, linking molecular strategies to host-level immune outcomes and clinical disease along a continuous host journey.

Following dissemination through blood and lymphatic routes, *B. burgdorferi* shifts from systemic transit to tissue colonization, establishing persistent niches within diverse host organs. This phase reflects the coordinated engagement of spirochetal adhesins with tissue-specific extracellular matrix components, together with localized immune modulation that permits sustained residence within host tissues. Clinically, these colonization events correspond to manifestations such as erythema migrans, carditis, neuroborreliosis, and Lyme arthritis, but at the biological level they represent a finely tuned process of niche establishment shaped by local extracellular architecture and immune context. Within the journey framework used here, these tissues function as “scenic stops”: intermediate waypoints where the spirochete anchors, adapts and recalibrates before further dissemination or long-term persistence. Rather than encountering a single decisive barrier, *B. burgdorferi* traverses a series of tissue-specific checkpoints that collectively shape its downstream infection dynamics (**Figure 3**).

### 5.1 Adhesion-mediated tissue anchoring and extracellular matrix engagement

Tissue colonization by *B. burgdorferi* relies on a broad suite of adhesins that interact with diverse host ligands, including decorin, fibronectin, glycosaminoglycans, laminin, and integrins, allowing the spirochete to establish niches across joints, heart, bladder, skin, and other tissues (5, 57). This adhesin repertoire is both modular and functionally redundant: multiple adhesins (Dbp family, BBK32, P66, Lmp1 and related adhesins) often serve overlapping functions, such that the loss of a single adhesin produces tissue-specific deficits rather than complete loss of infectivity (5, 58, 59). Exceptions occur when adhesins are required during early invasion steps, as observed for P66 (49). Allelic variation further refines tissue tropism, with distinct DbpA variants conferring differential organ targeting even in isogenic backgrounds (60). Numerous plasmid-borne and poorly annotated ORFs also serve to contribute, as demonstrated by transposon or deletion mutants that frequently exhibit defects in dissemination or reduced tissue burdens, thereby underscoring the breadth of the colonization machinery (61, 62). Temporal and environmental regulation adds another layer of complexity. OspC is essential immediately following transmission but is later downregulated; P66 functions prominently during vascular exit; and DbpA and Lmp1 contribute more substantially to later tissue burdens (5, 63). Gene requirements also vary according to the route of infection, with some genes critical during natural tick transmission (e.g., *bba64*) yet dispensable during needle inoculation, whereas others, such as *dbpBA*, remain effective during tick-mediated infection (64). Together, these layers define colonization as a multistep process in which adhesin-mediated extracellular matrix engagement represents one strategic axis within the spirochete’s broader survival portfolio. These physical interactions provide the architectural foundation upon which additional immune-modulatory and metabolic adaptations are later built.

### 5.2 Innate immune conditioning within tissue niches

Upon tissue entry, spirochetes encounter dense, organ-specific innate immune networks that shape early survival and inflammatory tone. These networks are composed of complement activity at tissue interfaces, resident macrophages, recruited monocytes and neutrophils, and pattern-recognition pathways that differ substantially across organs. Recent *in vivo* imaging and transcriptomics now allow these maneuvers to be mapped to specific tissues and early time points along the infection trajectory (59). Lyme disease Borreliae deploy a diversified suite of complement regulator-acquiring surface proteins (CRASPs) and other outer surface proteins that bind plasminogen, factor H, FHL-1, CFHR and C4b-binding protein, attenuating classical, lectin, and alternative pathway activation at tissue surfaces (5). This complement-evasion repertoire is both multifunctional and redundant: proteins including CspA/CspZ, OspE/OspF paralogs, BBK32, OspA, OspC, ErpA, ErpC, ErpP and BBA70 contribute simultaneously to serum resistance, vascular adhesion, and tissue tropism, with subtle sequence variation tuning host specificity and niche preference (13, 65, 66). Recent work highlights how individual surface proteins integrate ECM binding with complement inhibition, reinforcing the idea that colonization and immune evasion are mechanistically intertwined rather than separable modules (67). Beyond immediate complement resistance, newer studies indicate that *B. burgdorferi* actively reshape local innate immune tone. In Lyme carditis, localized interactions between spirochetes and macrophages induce durable changes in bacterial uptake and inflammatory responses with consequences that extend across the heart tissue and determine disease severity (68). Parallel studies reported early transcriptional re-programming of macrophages toward suppressed inflammatory states, suggesting that colonized tissues are not passive environments but are actively conditioned by the pathogen (69). A major advance at the tissue level is the dissection of type I interferon (IFN-I) response critical for the development of Lyme arthritis. Innate inflammatory set-points are further shaped by nucleic-acid sensing pathways. *In vivo*, activation of the cGAS–STING axis and recognition of c-di-AMP drive type I interferon responses with measurable effects on joint inflammation and broader tissue pathology (70, 71).

### 5.3 Adaptive immune remodeling in colonized tissues

Tissue-level transcriptomic profiling during *B. burgdorferi* infection has begun to resolve how these immune circuits diverge across organs (59). In skin, early infection is dominated by interferon signaling, with IFN-γ/IFN-I–rich microenvironments recruiting both innate and adaptive immune cells (72). Single-cell analyses of erythema migrans lesions revealed prominent IgM-expressing memory B cells engaged in antigen presentation and early antibody production, supporting a predominantly extrafollicular B-cell response (73). Consistent with these findings, lymph-node profiling indicates preferential extrafollicular B-cell activation rather than robust germinal-center responses during early dissemination (74).

Early tissue colonization is accompanied by profound remodeling of adaptive immune architecture. More recent work adds a tissue-linked layer of immune disruption: active distortion of B cell differentiation pathways within lymphoid organs (74). Single-cell RNA sequencing reveals a strongly extrafollicular B cell program early after infection, marked by robust plasmablast expansion, skewed class switching toward IgG3/IgG2b, and incomplete or aberrant germinal- center formation (74). Complementary functional studies demonstrate that germinal centers that do arise are transient, appearing by approximately day 15 in murine lymph nodes and spleen and collapsing by day 30, coincident with accumulation of short-lived plasmablasts and disruption of classic germinal-center architecture (75, 76). Together, these findings support a model in which *B. burgdorferi* converts lymphoid “rest stops” into sites of rapid but poorly organized antibody production: sufficient to blunt bacteremia and overt pathology in reservoir hosts, yet insufficient to generate high-affinity, long-lived neutralizing responses capable of clearing deeply embedded tissue populations. Recent analyses of *Borrelia*-specific antibody profiles and complement activity in human cohorts further refine this picture (65). Distinct serologic patterns, including isotype skewing and incomplete opsonizing capacity, have been linked to clinical stage and outcome (77). Notably, several outer surface proteins implicated in both tissue adhesion and complement evasion (e.g. CspZ and OspC variants) emerge repeatedly as immunodominant yet immunomodulatory antigens (65, 77). These findings reinforce the concept that *B. burgdorferi* actively steers B-cell responses toward non-sterilizing extrafollicular trajectories shaped by local tissue environments. These early disruptions of adaptive immune architecture foreshadow the antigenic remodeling strategies that later support prolonged tissue residency.

### 5.4 Genotype-dependent tissue tropism

Consistent with this framework, adaptive responses can establish a state of concomitant immunity: partial protection against reinfection with the same strain while permitting continued survival of pre-existing intratissue populations (5, 59). From a journey perspective, each colonized site becomes a provisional “safe house”, where local immune responses are reshaped into a pattern that limits tissue damage while tolerating low-level spirochetal presence. This immunological accommodation establishes permissive tissue niches that support early tissue persistence and shape subsequent immune trajectories.

Pre-existing polymorphisms in surface proteins such as OspC further influence which tissue “stops” the spirochete reaches most efficiently (5). Field and experimental studies in reservoir hosts extend this concept to organ-level patterns (78). OspC genotypes are unevenly distributed across organs, with certain variants preferentially recovered from joints, heart, or bladder samples compared with skin, consistent with genotype-specific tissue tropism and differential success in particular immune microenvironments (5, 50, 78). Comparative genomic analyses of invasive human isolates further demonstrate that a linked genetic module encompassing lp28–1 and lp56 correlates with increased rate of dissemination in highly virulent OspC type A strains (79). Thus, from the perspective of the journey, OspC act as a “route selector,” biasing access to specific tissue destinations. As tissue residency stabilizes, escalating adaptive immune pressure necessitates a shift from colonization toward long-term persistence, marking the transition into the extended stay.

## 6 Extended stay: chronic persistence and immune stalemate

The transition into this phase marks the final leg of the immunological journey, which is not a triumphant “arrival” but a prolonged negotiation among persistence, clearance, and immune control. In Lyme disease, this “extended stay” encompasses two broad, partially overlapping outcomes: (i) persistent infection or long-lived tissue colonization, most evident in untreated or inadequately treated hosts, and (ii) post-treatment states, including post-infectious Lyme arthritis and post-treatment Lyme disease symptoms or syndrome (PTLD[S]/PTLDS), in which fatigue, pain, cognitive complaints, or other symptoms persist for months or longer after standard therapy (80–83). Importantly, this phase is not defined by a single biological endpoint but by convergent mechanistic trajectories, ranging from continued microbial presence in protected niches to immune programs that persist after microbial clearance. Careful separation of active infection from post-infectious immune sequelae is therefore essential. A defining feature of persistence in *B. burgdorferi* is the failure of adaptive immunity to constrain the pathogen to a single antigenic identity, creating strong selection for antigenic remodeling (**Figure 3**).

### 6.1 Changing the façade: antigenic variation during tissue residency

As *B. burgdorferi* establishes sustained residency within tissues such as skin, synovium, heart and neural tissues, it encounters escalating adaptive immune surveillance. At this stage of the journey, continued occupancy depends on the ability of the pathogen to repeatedly revise its surface identity - an immunological “shape-shifting” events achieved through antigenic variation. The core engine of this process is the *vls* locus on linear plasmid lp28-1, long recognized as central to immune evasion (12, 84). *B. burgdorferi* strains lacking *vlsE* or critical components of the *vls* recombination machinery establish early infection but fail to persist beyond two weeks, coinciding with the emergence of an effective adaptive immune response (85–87). Recent methodological advances have enabled direct genetic manipulation of the *vls* locus, overcoming longstanding technical barriers and opening the system to detailed mechanistic interrogation (88). Long-read sequencing and recombination mapping reveal extraordinary *in vivo* diversification, generating tens of thousands of unique VlsE mosaics within a single infected host (89). Genetic dissection of DNA repair machinery further demonstrates that the MutL endonuclease activity is required for efficient *vlsE* recombination and, consequently, for persistence in immunocompetent mice (90), complementing earlier evidence implicating resolvases such as RuvAB (91). Together, these findings support a model in which *vls* locus functions as a privileged substrate for specialized recombination and repair machinery during mammalian infection. Comparative genomics shows that *vls* systems are conserved across all Lyme disease *Borrelia*, yet vary markedly in cassette number, organization, and sequence space, suggesting lineage-specific tuning to host range and ecological niche (92–94). Structural and immunologic analyses indicate that dominant antibody responses preferentially target relatively conserved internal regions such as IR6, which may act as decoy epitopes, while surface-exposed variable domains continually shift to prevent effective opsonization (95). More recently, VlsE has been shown to shield an arthritis-associated surface antigen from antibody binding, adding an epitope-masking layer to classical antigenic variation and directly linking the *vls* system to joint colonization and Lyme arthritis pathogenesis (96). Consistent with this multifunctionality, VlsE can partially complement the role of OspC during joint invasion and colonization in immunodeficient mice, positioning this lipoprotein as a nexus integrating antigenic variation, immune evasion, and tissue engagement (45). Recent studies further demonstrate that *vlsE* expression is subject to regulatory control *in vivo*, with YebC functioning as a transcriptional modulator that contributes to immune evasion and fitness in immunocompetent hosts (97). Thus, continued diversification at the *vls* locus enables ongoing remodeling of VlsE during mammalian infection, permitting prolonged survival under humoral immune pressure without dominance of a single antigenic variant. While antigenic variation enables prolonged residency under adaptive immune pressure, additional layers of innate immune containment shape whether persistence progresses toward microbial survival or post-infectious immune pathology. Complement evasion remains an important contributor at tissue interfaces and vascular boundaries, with multiple borrelial surface proteins mediating factor H acquisition to limit complement-mediated damage during dissemination and persistent infection (98–100). However, recent work emphasizes redundancy and context dependence within these systems: individual factor H-binding proteins do not consistently predict serum resistance or infectivity across experimental systems, cautioning against attributing persistence to any single complement evasion mechanism (101). Notably, this complement-facing biology is increasingly being leveraged translationally, as structure-guided modification of CspZ variants lacking factor H-binding capacity has been explored to enhance bactericidal responses and vaccine antigen performance (102, 103). Together, these findings underscore a central theme of the extended stay: persistence reflects layered, partially overlapping strategies whose relative importance shifts across tissues and stages of infection.

### 6.2 Antigen persistence and post-infectious inflammation

A major advance supporting an “immune-stalemate” model has been the recognition that persistent antigens derived from *B. burgdorferi*, particularly peptidoglycan, can function as durable inflammatory stimuli. Building on earlier evidence of peptidoglycan persistence in patients with Lyme arthritis (104), recent studies show that borrelial peptidoglycan can distribute and persist *in vivo* after antimicrobial therapy, providing a plausible substrate for post-infectious inflammation (105). The recent demonstration that polymeric borrelial peptidoglycan persists within liver and joint macrophages, reshaping systemic immune and metabolic signatures, underscores how antigen retention within tissue “safe houses” may continue to broadcast danger signals long after viable bacteria become sparse, potentially contributing to chronic symptomatology in a subset of hosts (106). Further mechanistic refinement reveals that both host and bacterial enzymes modify borrelial peptidoglycan fragments, shaping their immunostimulatory properties and mechanistically linking antigen persistence to sustained inflammatory signaling (105). This antigen-persistence axis offers a compelling explanation for antibiotic-refractory Lyme arthritis, in which synovial inflammation persists despite effective clearance of replicating organisms and is best understood as a post-infectious immune process rather than ongoing infection (107, 108). Consistent with this framework, impaired activation of immune resolution programs further contributes to chronic joint inflammation even after microbial clearance (109).

### 6.3 Phenotypic heterogeneity and antimicrobial tolerance

A complementary dimension of the extended stay is phenotypic heterogeneity under antimicrobial stress. Experimental studies indicate that *B. burgdorferi* populations can adopt antimicrobial-tolerant persister states and pleomorphic morphologies *in vitro*, including proposed viable-but-non-culturable (VBNC)-like forms (14, 110). In animal models, borrelial nucleic acids may be detected in tissues for months following antibiotic treatment despite the absence of culturable organisms (80). However, antimicrobial-tolerant persisters have been robustly demonstrated primarily *in vitro*, and the inability to recover viable bacteria from treated animals argues against the widespread persistence of resuscitable cells *in vivo* (80). Thus, the existence and clinical relevance of such persister populations during mammalian infection remain debated. Similarly, although biofilm-like growth has been explored in *B. afzelii* and *B. garinii* under laboratory conditions, the existence of true biofilms during mammalian infection remains controversial and lacks convincing *in vivo* evidence (111). Within an immune-stalemate framework, these phenotypes are therefore best viewed as contributors to transient antimicrobial tolerance rather than primary drivers of post-treatment disease.

### 6.4 Post-treatment immune dysregulation and PTLDS

Beyond joint pathology, immune dysregulation can persist systemically, and a subset of patients, estimated at approximately 10 to 20 percent, experience prolonged fatigue, pain, cognitive complaints, or other symptoms following standard therapy, collectively described as PTLDS (14). Contemporary reviews emphasize the heterogeneity of these presentations and caution against a unifying mechanism, instead implicating immune dysregulation, altered neuroimmune signaling, and maladaptive inflammatory set-points in the absence of active infection reflecting ongoing debate regarding the biological basis of PTLDS (80–82). Cohort-based studies further support associations between persistent symptoms and lingering immune signatures despite apparent microbial clearance (83, 112). Importantly, randomized clinical trials and systematic analyses consistently demonstrate no sustained benefit of prolonged antibiotic therapy for PTLDS, reinforcing the need to distinguish persistent symptoms from microbial persistence in both clinical management and mechanistic investigation (80, 82, 110).

Taken together, these observations position the extended stay not simply as a failure of immunity or therapy, but as a biological stalemate. In some contexts, *B. burgdorferi* persists at low levels within permissive niches; in others, the pathogen has been cleared, yet its molecular remnants and the immune programs they provoke continue to shape disease. By contrast, reservoir hosts tolerate persistent infection with minimal immunopathology, underscoring species-specific immune adaptations. Disentangling viable bacterial survival, antigen persistence, and prolonged immune aftereffects remains a central challenge for the field and a subject of active debate, with important implications for diagnostics, therapeutic strategies, and the interpretation of chronic Lyme disease manifestations. Across stages, infection follows a recognizable arc, marked by early innate suppression, tissue-level immune conditioning, derailment of germinal center maturation, and eventual immune stalemate. This trajectory reframes Lyme disease not simply as failed microbial clearance but as progressive immune reprogramming. These cumulative immune imprints ultimately surface in the clinic, where underlying biology must be translated into diagnosis and care.

## 7 The doctor’s office: clinical recognition and intervention

The clinical encounter represents the final, human-centered destination of the immunological journey of *B. burgdorferi*. In the doctor’s office, medicine must reconstruct a complex itinerary of ecological exposure, vector transmission, dissemination, and tissue colonization, compressing it into a moment of clinical pattern recognition. This stage becomes an active battleground where diagnostic limitations confront the spirochete’s evolved strategies for stealth. What begins on the forest floor ultimately presents as discrete clinical syndromes-erythema migrans (EM), early disseminated neurologic or cardiac involvement, or late inflammatory arthritis, each reflecting both the pathogen’s prior tissue tropism and the host’s evolving immune landscape.

### 7.1 Serologic diagnostics and clinical pattern recognition

Diagnosis of Lyme disease remains largely an exercise in *post hoc* immunological interpretation, relying primarily on host-derived antibodies rather than direct pathogen detection. In patients presenting with EM and compatible exposure histories, clinical diagnosis is sufficient and treatment should not be delayed, as serologic assays are often negative during this early window of immune evasion (113; Association of Public Health Laboratories (APHL): Suggested Reporting Language, Interpretation and Guidance Regarding Lyme Disease Serologic Test Results https://stacks.cdc.gov/view/cdc/156015). Current guidelines favor Modified Two-Tier Testing (MTTT), employing sequential or concurrent enzyme immunoassays, over traditional Standard Two-Tier Testing (STTT) (113);, improving early sensitivity and simplifying workflows (114–117). Nevertheless, dependence on adaptive immune responses imposes inherent limitations: a diagnostic window of seronegativity persists during early dissemination, while antibodies may remain detectable long after microbial clearance, complicating distinction between active disease and immunologic memory (118). Clinical diagnostics therefore represent retrospective reconstruction of immune history rather than direct snapshots of microbial burden. Clinical decision-making is further challenged by symptom overlap with autoimmune and post-infectious inflammatory conditions, underscoring the importance of pretest probability and staged interpretation. In this context, domestic animals, particularly dogs, serve as valuable sentinels of local transmission intensity. Canine seroprevalence often reflects changes in tick exposure earlier than human case detection, providing actionable epidemiologic context to refine clinical suspicion in endemic regions (119, 120). Emerging serologic platforms seek to improve this immunological snapshot through multiplex and scaffolded multi-epitope assays designed to better capture early B-cell responses (121, 122), as well as single-tier hybrid ELISA formats showing promise in early infection (123).

### 7.2 Direct detection and host-derived biomarkers

Direct pathogen detection, finding the traveler rather than the stamp, remains challenging because bacteremia in *B. burgdorferi* infection is typically low and transient (APHL: https://stacks.cdc.gov/view/cdc/156015). Advances in digital droplet PCR (ddPCR) have improved sensitivity of detecting *B. burgdorferi* DNA in cerebrospinal fluid, offering more definitive organism-level diagnosis in neuroborreliosis (124, 125), while synovial fluid PCR remains the diagnostic gold standard for confirming the “scenic stop” of Lyme arthritis (113, 118). Given these constraints, attention has increasingly shifted toward profiling host immune states rather than the pathogen itself. Metabolomic, proteomic, and transcriptomic profiling have identified biosignatures distinguishing early Lyme disease (126–128) and neuroborreliosis, including CXCL13 (129, 130), IL-10, TNF, and CCL8, offering a framework for immune-informed diagnostics and treatment monitoring (131–133).

The growing complexity of host-derived datasets has accelerated integration of artificial intelligence and machine learning into Lyme disease diagnostics, supporting biomarker discovery, proteomic interpretation, and explainable diagnostic frameworks (134). Parallel advances in microfluidics and smartphone-compatible platforms point toward future point-of-care tools capable of detecting antigens or host signatures during the critical early seronegative window (135–137). Although promising, these technologies require rigorous validation across diverse clinical settings and real-world cohorts before routine adoption.

### 7.3 Therapeutic management and prevention strategies

In clinical practice, diagnosis rapidly translates into therapeutic intervention. Current guidelines emphasize symptom-based, time-limited antimicrobial therapy tailored to disease stage and organ involvement, with early treatment aimed at preventing systemic dissemination and durable tissue colonization (113). Current standard-of-care therapy typically consists of short courses of doxycycline, amoxicillin, cefuroxime axetil, or azithromycin for early disease, with intravenous ceftriaxone, cefotaxime, and penicillin G reserved for selected neurologic or cardiac manifestations (113). From an immunological perspective, timely antibiotic therapy aims to resolve inflammatory triggers before they escalate into chronic dysregulation. However, a subset of patients develops persistent symptoms consistent with PTLDS, a state increasingly understood as a maladaptive immune legacy rather than ongoing infection, highlighting the clinical challenge of managing host responses that outlast pathogen clearance.

The most definitive intervention is prevention of the immune encounter altogether. The leading vaccine candidate, multivalent OspA-based vaccine VLA15 (Pfizer-Valneva), employs a transmission-blocking strategy in which vaccine-induced antibodies neutralize *B. burgdorferi* within the feeding tick midgut, preventing transmission to the human host. With Phase III trials (VALOR) fully enrolled and booster immunogenicity and safety data reported through late 2025, VLA15 represents the most advanced effort to shift Lyme disease management from downstream treatment to upstream interception (138). Parallel reservoir-targeted OspA vaccination strategies have demonstrated reductions in tick infection prevalence under field conditions, highlighting complementary approaches to lowering human exposure risk (139).

Additional preclinical strategies include mRNA-based vaccines encoding *Borrelia* antigens, e.g., OspA-encoding mRNA-lipid nanoparticle (140), OspC (141), other borrelial candidates as BBI39 (142, 143), viral-vector platforms (e.g., intranasal PIV5-based vaccines) (144, 145), passive immunization with long-acting anti-OspA monoclonal antibodies (https://www.idsociety.org/science-speaks-blog/2026/tick-borne-disease-vaccines-what-clinicians-should-know-in-2026/ Accessed Feb 10, 2026; https://www.umassmed.edu/news/news-archives/2022/05/massbiologics-research-into-preventive-shot-for-lyme-disease-continues-to-move-forward/ Accessed Feb 10, 2026), and multiple tick saliva antigens to induce a form of “tick immunity” (146). Collectively, these efforts reflect a multipronged translational pipeline aimed at reducing disease burden by targeting both pathogen and vector biology.

Together, advances in diagnostics, immunoprofiling, and prevention underscore that the doctor’s office is not merely the endpoint of infection, but a site where ecological exposure, microbial strategy, and host immunity converge into actionable clinical decisions. As diagnostic tools evolve from serology toward immune-state and organism-level detection, Lyme disease care is moving toward earlier recognition, mechanistic stratification, and more precision intervention. In this sense, the journey from forest floor to clinic comes full circle - not with closure, but with opportunity to intercept infection sooner, tailor treatment more intelligently, and ultimately transform Lyme disease from a stealthy ecological invader into a clinically manageable event.

## 8 Conclusions and future directions

While the clinical journey of Lyme disease culminates in the doctor’s office, the biological journey of *Borrelia burgdorferi* continues uninterrupted within its enzootic cycle. Host immune responses often constrain spirochete burden and resolve overt disease, yet long-term persistence in reservoir hosts enables re-acquisition by ticks and sustained transmission. Viewed through an immunological lens, Lyme disease emerges as a prolonged dialogue between pathogen and host. This co-evolutionary accommodation permits microbial survival within immunological gray zones, where clearance gives way to coexistence, revealing persistence not as immune failure but as the outcome of negotiated equilibrium between host defenses and pathogen adaptation. Defying classical Gram-negative paradigms, *B. burgdorferi* has evolved a distinct biological strategy characterized by transient bacteremia, tissue residency, antigenic remodeling, and immune recalibration favoring persistence over explosive expansion.

Despite substantial advances in defining the molecular and cellular mechanisms underlying dissemination, colonization, and immune evasion, fundamental questions remain regarding the determinants of immune resolution versus tolerance, tissue-specific persistence, and failure of durable immune memory. Emerging approaches in spatial biology, intravital imaging, multi-omics, and systems immunology now offer unprecedented opportunities to resolve host-pathogen interactions in real time and in tissue context. These technologies promise to move the field beyond static snapshots toward a dynamic understanding of infection as a shifting equilibrium among innate activation, adaptive remodeling, and tolerance-like immune states.

Future progress will require approaches that bridge scales that have traditionally been studied in isolation. Experimental models that better capture the full ecological transmission cycle, including tick-mediated infection and reservoir host dynamics, will be essential for understanding how immune responses shape pathogen fitness in natural settings. At the tissue level, spatial and single-cell multi-omics approaches offer new opportunities to resolve host-pathogen interactions within the microanatomical niches that support spirochete persistence. Translational advances may emerge from these insights, including next-generation vaccine strategies as well as standardized biomarker frameworks to better characterize post-treatment symptoms following infection. Integrating these ecological, immunological, and translational perspectives will be critical for transforming our understanding of Lyme disease from a linear infection model into a dynamic, multi-host biological system.

Ultimately, the central lesson of *B. burgdorferi* is not simply how a spirochete spreads, but how adaptive immunity can be diverted from sterilizing protection toward long-term accommodation. Deciphering the mechanisms that govern this immune stalemate across ecological, anatomical, and temporal scales will be essential for improving diagnostics, refining therapeutic strategies, and developing effective preventive interventions. In this light, words attributed to Louis Pasteur still resonate: “Gentlemen, it is the microbes who will have the last word (Messieurs, c’est les microbes qui auront le dernier mot)” (147). Spoken more than a century ago, they remain unnervingly relevant today, reminding us that understanding microbial strategies of immune navigation is essential if we hope to reclaim the final word.

## References

[1] Steere AC Grodzicki RL Kornblatt AN Craft JE Barbour AG Burgdorfer W . The spirochetal etiology of Lyme disease. N Engl J Med. (1983) 308:733–40. doi: 10.1056/nejm198303313081301. PMID: 6828118

[2] Steere AC . Lyme disease. N Engl J Med. (2001) 345:115–25. doi: 10.1016/0753-3322(91)90137-i 11450660

[3] Adeolu M Gupta RS . A phylogenomic and molecular marker based proposal for the division of the genus Borrelia into two genera: the emended genus Borrelia containing only the members of the relapsing fever Borrelia, and the genus Borreliella gen. nov. containing the members of the Lyme disease Borrelia (Borrelia burgdorferi sensu lato complex). Antonie van Leeuwenhoek. (2014) 105:1049–72. doi: 10.1007/s10482-014-0164-x. PMID: 24744012

[4] Fraser CM Casjens S Huang WM Sutton GG Clayton R Lathigra R . Genomic sequence of a Lyme disease spirochaete, Borrelia burgdorferi. Nature. (1997) 390:580–6. doi: 10.1038/37551. PMID: 9403685

[5] Strnad M Rudenko N Rego ROM . Pathogenicity and virulence of Borrelia burgdorferi. Virulence. (2023) 14:2265015. doi: 10.1080/21505594.2023.2265015. PMID: 37814488 PMC10566445

[6] Bourgeois JS Hu LT . Hitchhiker's guide to Borrelia burgdorferi. J Bacteriol. (2024) 206:e0011624. doi: 10.1128/jb.00116-24. PMID: 39140751 PMC11411949

[7] Radolf JD Caimano MJ Stevenson B Hu LT . Of ticks, mice and men: understanding the dual-host lifestyle of Lyme disease spirochaetes. Nat Rev Microbiol. (2012) 10:87–99. doi: 10.1038/nrmicro2714. PMID: 22230951 PMC3313462

[8] Steere AC Malawista SE Snydman DR Shope RE Andiman WA Ross MR . Lyme arthritis: an epidemic of oligoarticular arthritis in children and adults in three connecticut communities. Arthritis Rheum. (1977) 20:7–17. doi: 10.1002/art.1780200102. PMID: 836338

[9] Levi T Kilpatrick AM Mangel M Wilmers CC . Deer, predators, and the emergence of Lyme disease. Proc Natl Acad Sci USA. (2012) 109:10942–7. doi: 10.1073/pnas.1204536109. PMID: 22711825 PMC3390851

[10] Ostfeld RS Levi T Keesing F Oggenfuss K Canham CD . Tick-borne disease risk in a forest food web. Ecology. (2018) 99:1562–73. doi: 10.1002/ecy.2386. PMID: 29738078

[11] Pal U Li X Wang T Montgomery RR Ramamoorthi N Desilva AM . TROSPA, an Ixodes scapularis receptor for Borrelia burgdorferi. Cell. (2004) 119:457–68. doi: 10.1016/j.cell.2004.10.027. PMID: 15537536

[12] Norris SJ . vls antigenic variation systems of Lyme disease Borrelia: eluding host immunity through both random, segmental gene conversion and framework heterogeneity. Microbiol Spectr. (2014) 2. doi: 10.1128/microbiolspec.MDNA3-0038-2014. PMID: 26104445 PMC4480602

[13] Lin YP Diuk-Wasser MA Stevenson B Kraiczy P . Complement evasion contributes to Lyme borreliae-host associations. Trends Parasitol. (2020) 36:634–45. doi: 10.1016/j.pt.2020.04.011. PMID: 32456964 PMC7292789

[14] Cabello FC Embers ME Newman SA Godfrey HP . Borreliella burgdorferi antimicrobial-tolerant persistence in Lyme disease and posttreatment Lyme disease syndromes. mBio. (2022) 13:e0344021. doi: 10.1128/mbio.03440-21. PMID: 35467428 PMC9239140

[15] Wolcott KA Margos G Fingerle V Becker NS . Host association of Borrelia burgdorferi sensu lato: a review. Ticks Tick-borne Dis. (2021) 12:101766. doi: 10.1016/j.ttbdis.2021.101766. PMID: 34161868

[16] Barthold SW . Specificity of infection-induced immunity among Borrelia burgdorferi sensu lato species. Infect Immun. (1999) 67:36–42. doi: 10.1128/iai.67.1.36-42.1999. PMID: 9864193 PMC96274

[17] Steere AC Strle F Wormser GP Hu LT Branda JA Hovius JW . Lyme borreliosis. Nat Rev Dis Primers. (2016) 2:16090. doi: 10.1128/9781555816490.ch11. PMID: 27976670 PMC5539539

[18] Kurokawa C Lynn GE Pedra JHF Pal U Narasimhan S Fikrig E . Interactions between Borrelia burgdorferi and ticks. Nat Rev Microbiol. (2020) 18:587–600. doi: 10.1038/s41579-020-0400-5. PMID: 32651470 PMC7351536

[19] Gardner AM Pawlikowski NC Hamer SA Hickling GJ Miller JR Schotthoefer AM . Landscape features predict the current and forecast the future geographic spread of Lyme disease. Proc Biol Sci. (2020) 287:20202278. doi: 10.1098/rspb.2020.2278. PMID: 33352074 PMC7779494

[20] Ogden NH Ben Beard C Ginsberg HS Tsao JI . Possible effects of climate change on ixodid ticks and the pathogens they transmit: predictions and observations. J Med Entomol. (2021) 58:1536–45. doi: 10.1093/jme/tjaa220. PMID: 33112403 PMC9620468

[21] Diuk-Wasser MA VanAcker MC Fernandez MP . Impact of land use changes and habitat fragmentation on the eco-epidemiology of tick-borne diseases. J Med Entomol. (2021) 58:1546–64. doi: 10.1093/jme/tjaa209. PMID: 33095859

[22] Deshpande G Beetch JE Heller JG Naqvi OH Kuhn KG . Assessing the influence of climate change and environmental factors on the top tick-borne diseases in the United States: a systematic review. Microorganisms. (2023) 12. doi: 10.3390/microorganisms12010050. PMID: 38257877 PMC10821204

[23] Westcott JR Bowden JJ Savage J Doody KM . Rapid northward expansion of the Blacklegged Tick, Ixodes scapularis, in response to climate change. Glob Chang Biol. (2025) 31:e70591. doi: 10.1111/gcb.70591. PMID: 41195696 PMC12590538

[24] Kleissl L Weninger S Winkler F Ruivo M Wijnveld M Strobl J . Ticks' tricks: immunomodulatory effects of ixodid tick saliva at the cutaneous tick-host interface. Front Immunol. (2025) 16:1520665. doi: 10.3389/fimmu.2025.1520665. PMID: 40213541 PMC11983607

[25] Šimo L Kazimirova M Richardson J Bonnet SI . The essential role of tick salivary glands and saliva in tick feeding and pathogen transmission. Front Cell Infect Microbiol. (2017) 7:281. doi: 10.3389/fcimb.2017.00281, PMID: 28690983 PMC5479950

[26] Kim TK Tirloni L Pinto AF Moresco J Yates JR da Silva Vaz I . Ixodes scapularis tick saliva proteins sequentially secreted every 24 h during blood feeding. PloS NeglTrop Dis. (2016) 10:e0004323. doi: 10.1371/journal.pntd.0004323. PMID: 26751078 PMC4709002

[27] Kim TK Tirloni L Bencosme-Cuevas E Kim TH Diedrich JK Yates JR . Borrelia burgdorferi infection modifies protein content in saliva of Ixodes scapularis nymphs. BMC Genomics. (2021) 22:152. doi: 10.1186/s12864-021-07429-0. PMID: 33663385 PMC7930271

[28] Strobl J Mündler V Müller S Gindl A Berent S Schötta AM . Tick feeding modulates the human skin immune landscape to facilitate tick-borne pathogen transmission. J Clin Invest. (2022) 132. doi: 10.1172/jci161188. PMID: 36166299 PMC9621130

[29] Jin L Jiang BG Yin Y Guo J Jiang JF Qi X . Interference with LTβR signaling by tick saliva facilitates transmission of Lyme disease spirochetes. Proc Natl Acad Sci USA. (2022) 119:e2208274119. doi: 10.1073/pnas.2208274119. PMID: 36383602 PMC9704693

[30] Strobl J Kleissl L Eder J Connolly S Frey T Gail LM . Human epidermal Langerhans cells induce tolerance and hamper T cell function upon tick-borne pathogen transmission. Nat Commun. (2025) 16:11715. doi: 10.1038/s41467-025-66821-6. PMID: 41315257 PMC12753675

[31] Diuk-Wasser MA Vannier E Krause PJ . Coinfection by Ixodes tick-borne pathogens: ecological, epidemiological, and clinical consequences. Trends Parasitol. (2016) 32:30–42. doi: 10.1016/j.pt.2015.09.008. PMID: 26613664 PMC4713283

[32] Parveen N Bhanot P . Babesia microti-Borrelia burgdorferi coinfection. Pathogens. (2019) 8. doi: 10.3390/pathogens8030117. PMID: 31370180 PMC6789475

[33] Ssentongo P Venugopal N Zhang Y Chinchilli VM Ba DM . Beyond human babesiosis: prevalence and association of Babesia coinfection with mortality in the United States, 2015-2022: a retrospective cohort study. Open Forum Infect Dis. (2024) 11:ofae504. doi: 10.1093/ofid/ofae504. PMID: 39381028 PMC11460071

[34] Bao F Yang J Ji Z Zha C Liu A . Global prevalence of Borrelia burgdorferi, Anaplasma phagocytophilum, and Babesia microti coinfections in human populations from 1946 to 2024: a systematic review and meta-analysis. New Microbes New Infect. (2025) 67:101621. doi: 10.1016/j.nmni.2025.101621. PMID: 41050736 PMC12491743

[35] Rocha SC Moustafa MAM Velásquez CV Azuama OC Zafar K Meyer C . Long-term survival of Babesia microti and Borrelia burgdorferi in C3H/HeJ mice and their effect on Lyme arthritis and babesiosis manifestations. Microbiol Spectr. (2025) 13:e0025225. doi: 10.1128/spectrum.00252-25. PMID: 40793757 PMC12403850

[36] Grab DJ Nyarko E Barat NC Nikolskaia OV Dumler JS . Anaplasma phagocytophilum-Borrelia burgdorferi coinfection enhances chemokine, cytokine, and matrix metalloprotease expression by human brain microvascular endothelial cells. Clin Vaccine Immunol. (2007) 14:1420–4. doi: 10.1128/cvi.00308-07. PMID: 17898182 PMC2168173

[37] Raghunandanan S Zhang K Zhang Y Priya R Sze CW Lou Y . MCP5, a methyl-accepting chemotaxis protein regulated by both the Hk1-Rrp1 and Rrp2-RpoN-RpoS pathways, is required for the immune evasion of Borrelia burgdorferi. PloS Pathog. (2024) 20:e1012327. doi: 10.1371/journal.ppat.1012327. PMID: 39775665 PMC11723614

[38] Rego RO Bestor A Stefka J Rosa PA . Population bottlenecks during the infectious cycle of the Lyme disease spirochete Borrelia burgdorferi. PloS One. (2014) 9:e101009. doi: 10.1371/journal.pone.0101009. PMID: 24979342 PMC4076273

[39] Harman MW Dunham-Ems SM Caimano MJ Belperron AA Bockenstedt LK Fu HC . The heterogeneous motility of the Lyme disease spirochete in gelatin mimics dissemination through tissue. Proc Natl Acad Sci USA. (2012) 109:3059–64. doi: 10.1016/j.bpj.2011.11.829. PMID: 22315410 PMC3286914

[40] Guo Z Zhao N Chung TD Singh A Pandey I Wang L . Visualization of the dynamics of invasion and intravasation of the bacterium that causes Lyme disease in a tissue engineered dermal microvessel model. Adv Sci (Weinh). (2022) 9:e2204395. doi: 10.1002/advs.202204395. PMID: 36156464 PMC9762293

[41] Strnad M Týč J Kitzberger F Kopecká J Rego ROM Vancová M . Targeted volume imaging reveals early vascular interactions of Lyme disease pathogen in skin. Nat Commun. (2025) 16:9330. doi: 10.1038/s41467-025-64326-w. PMID: 41125572 PMC12546650

[42] Moriarty TJ Norman MU Colarusso P Bankhead T Kubes P Chaconas G . Real-time high resolution 3D imaging of the Lyme disease spirochete adhering to and escaping from the vasculature of a living host. PloS Pathog. (2008) 4:e1000090. doi: 10.1371/journal.ppat.1000090. PMID: 18566656 PMC2408724

[43] Norman MU Moriarty TJ Dresser AR Millen B Kubes P Chaconas G . Molecular mechanisms involved in vascular interactions of the Lyme disease pathogen in a living host. PloS Pathog. (2008) 4:e1000169. doi: 10.1371/journal.ppat.1000169. PMID: 18833295 PMC2542414

[44] Moriarty TJ Shi M Lin YP Ebady R Zhou H Odisho T . Vascular binding of a pathogen under shear force through mechanistically distinct sequential interactions with host macromolecules. Mol Microbiol. (2012) 86:1116–31. doi: 10.1111/mmi.12045. PMID: 23095033 PMC3508296

[45] Tan X Lin YP Pereira MJ Castellanos M Hahn BL Anderson P . VlsE, the nexus for antigenic variation of the Lyme disease spirochete, also mediates early bacterial attachment to the host microvasculature under shear force. PloS Pathog. (2022) 18:e1010511. doi: 10.1371/journal.ppat.1010511. PMID: 35605029 PMC9166660

[46] Bista S Singh P Bernard Q Yang X Hart T Lin YP . A novel laminin-binding protein mediates microbial-endothelial cell interactions and facilitates dissemination of Lyme disease pathogens. J Infect Dis. (2020) 221:1438–47. doi: 10.1093/infdis/jiz626. PMID: 31758693 PMC7137894

[47] Niddam AF Ebady R Bansal A Koehler A Hinz B Moriarty TJ . Plasma fibronectin stabilizes Borrelia burgdorferi-endothelial interactions under vascular shear stress by a catch-bond mechanism. Proc Natl Acad Sci USA. (2017) 114:E3490–8. doi: 10.1073/pnas.1615007114. PMID: 28396443 PMC5410840

[48] Tan X Petri B DeVinney R Jenne CN Chaconas G . The Lyme disease spirochete can hijack the host immune system for extravasation from the microvasculature. Mol Microbiol. (2021) 116:498–515. doi: 10.1111/mmi.14728. PMID: 33891779

[49] Kumar D Ristow LC Shi M Mukherjee P Caine JA Lee WY . Intravital imaging of vascular transmigration by the Lyme spirochete: requirement for the integrin binding residues of the B. burgdorferi P66 protein. PloS Pathog. (2015) 11:e1005333. doi: 10.1371/journal.ppat.1005333. PMID: 26684456 PMC4686178

[50] Lin YP Tan X Caine JA Castellanos M Chaconas G Coburn J . Strain-specific joint invasion and colonization by Lyme disease spirochetes is promoted by outer surface protein C. PloS Pathog. (2020) 16:e1008516. doi: 10.1371/journal.ppat.1008516. PMID: 32413091 PMC7255614

[51] Tan X Castellanos M Chaconas G . Choreography of Lyme disease spirochete adhesins to promote vascular escape. Microbiol Spectr. (2023) 11:e0125423. doi: 10.1128/spectrum.01254-23. PMID: 37255427 PMC10434219

[52] Yuste RA Muenkel M Axarlis K Gómez Benito MJ Reuss A Blacker G . Borrelia burgdorferi modulates the physical forces and immunity signaling in endothelial cells. iScience. (2022) 25:104793. doi: 10.1016/j.isci.2022.104793. PMID: 35992087 PMC9389243

[53] Alvarez-Olmedo D Kamaliddin C Verhey TB Ho M De Vinney R Chaconas G . Transendothelial migration of the Lyme disease spirochete involves spirochete internalization as an intermediate step through a transcellular pathway that involves Cdc42 and Rac1. Microbiol Spectr. (2025) 13:e0222124. doi: 10.1128/spectrum.02221-24. PMID: 39727396 PMC11792520

[54] Wang L Xia Z Singh A Murarka B Baumgarth N Aucott JN . Extravasation of Borrelia burgdorferi across the blood-brain barrier is an extremely rare event. Adv Sci (Weinh). (2025) 12:e2413199. doi: 10.1016/b978-012677530-3/50316-0. PMID: 40071764 PMC12061299

[55] Divan A Casselli T Narayanan SA Mukherjee S Zawieja DC Watt JA . Borrelia burgdorferi adhere to blood vessels in the dura mater and are associated with increased meningeal T cells during murine disseminated borreliosis. PloS One. (2018) 13:e0196893. doi: 10.1371/journal.pone.0196893. PMID: 29723263 PMC5933741

[56] Casselli T Divan A Vomhof-DeKrey EE Tourand Y Pecoraro HL Brissette CA . A murine model of Lyme disease demonstrates that Borrelia burgdorferi colonizes the dura mater and induces inflammation in the central nervous system. PloS Pathog. (2021) 17:e1009256. doi: 10.1371/journal.ppat.1009256. PMID: 33524035 PMC7877756

[57] Brissette CA Gaultney RA . That's my story, and I'm sticking to it--an update on B. burgdorferi adhesins. Front Cell Infect Microbiol. (2014) 4:41. doi: 10.3389/fcimb.2014.00041. PMID: 24772392 PMC3982108

[58] Caine JA Coburn J . Multifunctional and redundant roles of Borrelia burgdorferi outer surface proteins in tissue adhesion, colonization, and complement evasion. Front Immunol. (2016) 7:442. doi: 10.3389/fimmu.2016.00442. PMID: 27818662 PMC5073149

[59] Coburn J Garcia B Hu LT Jewett MW Kraiczy P Norris SJ . Lyme disease pathogenesis. Curr Issues Mol Biol. (2021) 42:473–518. doi: 10.21775/cimb.042.473. PMID: 33353871 PMC8046170

[60] Lin YP Benoit V Yang X Martínez-Herranz R Pal U Leong JM . Strain-specific variation of the decorin-binding adhesin DbpA influences the tissue tropism of the lyme disease spirochete. PloS Pathog. (2014) 10:e1004238. doi: 10.1371/journal.ppat.1004238. PMID: 25079227 PMC4117581

[61] Lin T Troy EB Hu LT Gao L Norris SJ . Transposon mutagenesis as an approach to improved understanding of Borrelia pathogenesis and biology. Front Cell Infect Microbiol. (2014) 4:63. doi: 10.3389/fcimb.2014.00063. PMID: 24904839 PMC4033020

[62] Crowley MA Bankhead T . Potential regulatory role in mammalian host adaptation for a small intergenic region of Lp17 in the Lyme disease spirochete. Front Cell Infect Microbiol. (2022) 12:892220. doi: 10.3389/fcimb.2022.892220. PMID: 35586252 PMC9108270

[63] Yang X Lin YP Heselpoth RD Buyuktanir O Qin J Kung F . Middle region of the Borrelia burgdorferi surface-located protein 1 (Lmp1) interacts with host chondroitin-6-sulfate and independently facilitates infection. Cell Microbiol. (2016) 18:97–110. doi: 10.1111/cmi.12487. PMID: 26247174 PMC4718918

[64] Gilmore RD Howison RR Dietrich G Patton TG Clifton DR Carroll JA . The bba64 gene of Borrelia burgdorferi, the Lyme disease agent, is critical for mammalian infection via tick bite transmission. Proc Natl Acad Sci USA. (2010) 107:7515–20. doi: 10.1073/pnas.1000268107. PMID: 20368453 PMC2867675

[65] Marcinkiewicz AL Brangulis K Dupuis AP Hart TM Zamba-Campero M Nowak TA . Structural evolution of an immune evasion determinant shapes pathogen host tropism. Proc Natl Acad Sci USA. (2023) 120:e2301549120. doi: 10.1073/pnas.2301549120. PMID: 37364114 PMC10319004

[66] Thomas S Schulz AM Leong JM Zeczycki TN Garcia BL . The molecular determinants of classical pathway complement inhibition by OspEF-related proteins of Borrelia burgdorferi. J Biol Chem. (2024) 300:107236. doi: 10.1016/j.jbc.2024.107236. PMID: 38552741 PMC11066524

[67] Powell-Pierce AD Booth CE Smith PG Shapiro BL Allen SS Garcia BL . BBK32 attenuates antibody-dependent complement-mediated killing of infectious Borreliella burgdorferi isolates. PloS Pathog. (2025) 21:e1013361. doi: 10.1371/journal.ppat.1013361. PMID: 40705830 PMC12316396

[68] Barriales D Martín-Ruiz I Carreras-González A Montesinos-Robledo M Azkargorta M Iloro I . Borrelia burgdorferi infection induces long-term memory-like responses in macrophages with tissue-wide consequences in the heart. PloS Biol. (2021) 19:e3001062. doi: 10.1371/journal.pbio.3001062. PMID: 33395408 PMC7808612

[69] Petnicki-Ocwieja T McCarthy JE Powale U Langston PK Helble JD Hu LT . Borrelia burgdorferi initiates early transcriptional re-programming in macrophages that supports long-term suppression of inflammation. PloS Pathog. (2023) 19:e1011886. doi: 10.1371/journal.ppat.1011886. PMID: 38157387 PMC10783791

[70] Farris LC Torres-Odio S Adams LG West AP Hyde JA . Borrelia burgdorferi engages mammalian type I IFN responses via the cGAS-STING pathway. J Immunol. (2023) 210:1761–70. doi: 10.4049/jimmunol.2200354. PMID: 37067290 PMC10192154

[71] Priya R Ye M Raghunanadanan S Liu Q Li W Yu Q . Borrelia burgdorferi c-di-AMP is a key extracellular pathogen-associated molecular pattern to elicit type I interferon responses in mammalian hosts. J Immunol. (2025) 214:2325–37. doi: 10.1093/jimmun/vkaf133. PMID: 40611506 PMC12481034

[72] Marques A Schwartz I Wormser GP Wang Y Hornung RL Demirkale CY . Transcriptome assessment of erythema migrans skin lesions in patients with early Lyme disease reveals predominant interferon signaling. J Infect Dis. (2017) 217:158–67. doi: 10.1093/infdis/jix563. PMID: 29099929 PMC5853807

[73] Jiang R Meng H Raddassi K Fleming I Hoehn KB Dardick KR . Single-cell immunophenotyping of the skin lesion erythema migrans identifies IgM memory B cells. JCI Insight. (2021) 6. doi: 10.1172/jci.insight.148035. PMID: 34061047 PMC8262471

[74] Rinne V Gröndahl-Yli-Hannuksela K Fair-Mäkelä R Salmi M Rantakari P Lönnberg T . Single-cell transcriptome analysis of the early immune response in the lymph nodes of Borrelia burgdorferi-infected mice. Microbes Infect. (2025) 27:105424. doi: 10.1016/j.micinf.2024.105424. PMID: 39306236

[75] Tracy KE Baumgarth N . Borrelia burgdorferi manipulates innate and adaptive immunity to establish persistence in rodent reservoir hosts. Front Immunol. (2017) 8:116. doi: 10.3389/fimmu.2017.00116. PMID: 28265270 PMC5316537

[76] Williams MT Zhang Y Pulse ME Berg RE Allen MS . Suppression of host humoral immunity by Borrelia burgdorferi varies over the course of infection. Infect Immun. (2024) 92:e0001824. doi: 10.1128/iai.00018-24. PMID: 38514468 PMC11003232

[77] Bowman KA Wiggins CD DeRiso E Paul S Strle K Branda JA . Borrelia-specific antibody profiles and complement deposition in joint fluid distinguish antibiotic-refractory from -responsive Lyme arthritis. iScience. (2024) 27:108804. doi: 10.1016/j.isci.2024.108804. PMID: 38303696 PMC10830897

[78] Zawada SG von Fricken ME Weppelmann TA Sikaroodi M Gillevet PM . Genetic variation of Borreliella burgdorferi in Fairfax County, Virginia, targeting the OspC gene in white-footed mice. Front Microbiol. (2022) 13:998365. doi: 10.3389/fmicb.2022.998365. PMID: 36466686 PMC9715758

[79] Lemieux JE Huang W Hill N Cerar T Freimark L Hernandez S . Whole genome sequencing of human Borrelia burgdorferi isolates reveals linked blocks of accessory genome elements located on plasmids and associated with human dissemination. PloS Pathog. (2023) 19:e1011243. doi: 10.1371/journal.ppat.1011243. PMID: 37651316 PMC10470944

[80] Marques A . Persistent symptoms after treatment of Lyme disease. Infect Dis Clin North Am. (2022) 36:621–38. doi: 10.1016/j.idc.2022.04.004. PMID: 36116839 PMC9494579

[81] Talbot NC Spillers NJ Luther P Flanagan C Soileau LG Ahmadzadeh S . Lyme disease and post-treatment Lyme disease syndrome: Current and developing treatment options. Cureus. (2023) 15:e43112. doi: 10.7759/cureus.43112. PMID: 37692614 PMC10483257

[82] Dersch R Torbahn G Rauer S . Treatment of post-treatment Lyme disease symptoms-a systematic review. Eur J Neurol. (2024) 31:e16293. doi: 10.1111/ene.16293. PMID: 38606630 PMC11235603

[83] Baarsma ME Hovius JW . Persistent symptoms after Lyme disease: Clinical characteristics, predictors, and classification. J Infect Dis. (2024) 230:S62–9. doi: 10.1093/infdis/jiae203. PMID: 39140720

[84] Zhang JR Hardham JM Barbour AG Norris SJ . Antigenic variation in Lyme disease borreliae by promiscuous recombination of VMP-like sequence cassettes. Cell. (1997) 89:275–85. doi: 10.1016/s0092-8674(02)09630-7. PMID: 9108482

[85] Rogovskyy AS Casselli T Tourand Y Jones CR Owen JP Mason KL . Evaluation of the importance of VlsE antigenic variation for the enzootic cycle of Borrelia burgdorferi. PloS One. (2015) 10:e0124268. doi: 10.1371/journal.pone.0124268. PMID: 25893989 PMC4404307

[86] Magunda PR Bankhead T . Investigating the potential role of non-vls genes on linear plasmid 28–1 in virulence and persistence by Borrelia burgdorferi. BMC Microbiol. (2016) 16:180. doi: 10.1186/s12866-016-0806-4. PMID: 27502325 PMC4977671

[87] Bankhead T Chaconas G . The role of VlsE antigenic variation in the Lyme disease spirochete: Persistence through a mechanism that differs from other pathogens. Mol Microbiol. (2007) 65:1547–58. doi: 10.1111/j.1365-2958.2007.05895.x. PMID: 17714442

[88] Singh P Bankhead T . Breaking a barrier: In trans vlsE recombination and genetic manipulation of the native vlsE gene of the Lyme disease pathogen. PloS Pathog. (2025) 21:e1012871. doi: 10.1371/journal.ppat.1012871. PMID: 39792948 PMC11756760

[89] Verhey TB Castellanos M Chaconas G . Analysis of recombinational switching at the antigenic variation locus of the Lyme spirochete using a novel PacBio sequencing pipeline. Mol Microbiol. (2018) 107:104–15. doi: 10.1111/mmi.13969. PMID: 29105221

[90] Castellanos M Verhey TB Goldstein M Chaconas G . The putative endonuclease activity of MutL is required for the segmental gene conversion events that drive antigenic variation of the Lyme disease spirochete. Front Microbiol. (2022) 13:888494. doi: 10.3389/fmicb.2022.888494. PMID: 35663861 PMC9159922

[91] Lin T Gao L Edmondson DG Jacobs MB Philipp MT Norris SJ . Central role of the Holliday junction helicase RuvAB in vlsE recombination and infectivity of Borrelia burgdorferi. PloS Pathog. (2009) 5:e1000679. doi: 10.1371/journal.ppat.1000679. PMID: 19997622 PMC2780311

[92] Li L Di L Akther S Zeglis BM Qiu W . Evolution of the vls antigenic variability locus of the Lyme disease pathogen and development of recombinant monoclonal antibodies targeting conserved VlsE epitopes. Microbiol Spectr. (2022) 10:e0174322. doi: 10.1128/spectrum.01743-22. PMID: 36150043 PMC9604149

[93] Norris SJ Brangulis K . Meta-analysis of the Vmp-like sequences of Lyme disease Borrelia: Evidence for the evolution of an elaborate antigenic variation system. Front Microbiol. (2024) 15:1469411. doi: 10.3389/fmicb.2024.1469411. PMID: 39450289 PMC11499132

[94] Kang H Choi YJ Park JY Lee K Jang WJ . Comparative genomics of two newly sequenced rodent-derived and one previously reported tick-derived Borrelia garinii strains from South Korea reveals plasmid variation and virulence gene diversity. Pathogens. (2025) 14. doi: 10.3390/pathogens14111182. PMID: 41305418 PMC12655509

[95] Embers ME Liang FT Howell JK Jacobs MB Purcell JE Norris SJ . Antigenicity and recombination of VlsE, the antigenic variation protein of Borrelia burgdorferi, in rabbits, a host putatively resistant to long-term infection with this spirochete. FEMS Immunol Med Microbiol. (2007) 50:421–9. doi: 10.1111/j.1574-695x.2007.00276.x. PMID: 17596185

[96] Lone AG Bankhead T . The Borrelia burgdorferi VlsE lipoprotein prevents antibody binding to an arthritis-related surface antigen. Cell Rep. (2020) 30:3663–3670.e5. doi: 10.1016/j.celrep.2020.02.081. PMID: 32187539 PMC7162589

[97] Zhang Y Chen T Raghunandanan S Xiang X Yang J Liu Q . YebC regulates variable surface antigen VlsE expression and is required for host immune evasion in Borrelia burgdorferi. PloS Pathog. (2020) 16:e1008953. doi: 10.1371/journal.ppat.1008953. PMID: 33048986 PMC7584230

[98] Dulipati V Meri S Panelius J . Complement evasion strategies of Borrelia burgdorferi sensu lato. FEBS Lett. (2020) 594:2645–56. doi: 10.1002/1873-3468.13894. PMID: 32748966

[99] Marcinkiewicz AL Lieknina I Yang X Lederman PL Hart TM Yates J . The factor H-binding site of cspZ as a protective target against multistrain, tick-transmitted lyme disease. Infect Immun. (2020) 88. doi: 10.1128/iai.00956-19. PMID: 32122944 PMC7171238

[100] Trezel P Guérin M Da Ponte H Maffucci I Octave S Avalle B . Borrelia surface proteins: new horizons in Lyme disease diagnosis. Appl Microbiol Biotechnol. (2025) 109:156. doi: 10.1007/s00253-025-13490-6. PMID: 40590992 PMC12213884

[101] Cramer NA Socarras KM Earl J Ehrlich GD Marconi RT . Borreliella burgdorferi factor H-binding proteins are not required for serum resistance and infection in mammals. Infect Immun. (2024) 92:e0052923. doi: 10.1128/iai.00529-23. PMID: 38289123 PMC10929407

[102] McKaig CW Malfetano J Tran Y Yang X Pal U Wycoff K . Complement therapeutic factor H-IgG proteins as pre-exposure prophylaxes against Lyme borreliae infections. J Immunol. (2025) 214:2663–75. doi: 10.1093/jimmun/vkaf195. PMID: 40847470 PMC12435995

[103] Brangulis K Sürth V Marcinkiewicz AL Akopjana I Kazaks A Bogans J . CspZ variant-specific interaction with factor H incorporates a metal site to support Lyme borreliae complement evasion. J Biol Chem. (2025) 301:108083. doi: 10.1016/j.jbc.2024.108083. PMID: 39675703 PMC11773018

[104] Jutras BL Lochhead RB Kloos ZA Biboy J Strle K Booth CJ . Borrelia burgdorferi peptidoglycan is a persistent antigen in patients with Lyme arthritis. Proc Natl Acad Sci USA. (2019) 116:13498–507. doi: 10.1073/pnas.1904170116. PMID: 31209025 PMC6613144

[105] McCausland JW Kloos ZA Irnov I Sonnert ND Zhou J Putnik R . Bacterial and host enzymes modulate the pro-inflammatory response elicited by the peptidoglycan of Lyme disease agent Borrelia burgdorferi. PloS Pathog. (2025) 21:e1013324. doi: 10.1371/journal.ppat.1013324. PMID: 40623106 PMC12279116

[106] McClune ME Ebohon O Dressler JM Davis MM Tupik JD Lochhead RB . The peptidoglycan of Borrelia burgdorferi can persist in discrete tissues and cause systemic responses consistent with chronic illness. Sci Transl Med. (2025) 17:eadr2955. doi: 10.1126/scitranslmed.adr2955. PMID: 40267217 PMC12207536

[107] Steere AC . Lyme arthritis: A 50-year journey. J Infect Dis. (2024) 230:S1–S10. doi: 10.1093/infdis/jiae126. PMID: 39140724 PMC11322885

[108] Sigal LH . Aspects of the immunopathogenesis of lyme arthritis. Microorganisms. (2025) 13. doi: 10.3390/microorganisms13071602. PMID: 40732111 PMC12300122

[109] Lochhead RB Strle K Arvikar SL Weis JJ Steere AC . Lyme arthritis: linking infection, inflammation and autoimmunity. Nat Rev Rheumatol. (2021) 17:449–61. doi: 10.1038/s41584-021-00648-5. PMID: 34226730 PMC9488587

[110] Baker PJ . A review of antibiotic-tolerant persisters and their relevance to posttreatment lyme disease symptoms. Am J Med. (2020) 133:429–31. doi: 10.1016/j.amjmed.2019.12.007. PMID: 31926865

[111] Fabrizio G Cavallo I Sivori F Truglio M Kovacs D Francalancia M . Genomic characterization and antibiotic susceptibility of biofilm-forming Borrelia afzelii and Borrelia garinii from patients with erythema migrans. Front Cell Infect Microbiol. (2025) 15:1619660. doi: 10.3389/fcimb.2025.1619660. PMID: 40692686 PMC12277364

[112] Hernández SA Ogrinc K Korva M Kastrin A Bogovič P Rojko T . Association of persistent symptoms after lyme neuroborreliosis and increased levels of interferon-α in blood. Emerg Infect Dis. (2023) 29:1091–101. doi: 10.3201/eid2906.221685, PMID: 37209716 PMC10202885

[113] Lantos PM Rumbaugh J Bockenstedt LK Falck-Ytter YT Aguero-Rosenfeld ME Auwaerter PG . Clinical practice guidelines by the infectious diseases society of america (IDSA), american academy of neurology (AAN), and american college of rheumatology (ACR): 2020 guidelines for the prevention, diagnosis and treatment of lyme disease. Clin Infect Dis. (2021) 72:e1–e48. doi: 10.1093/cid/ciaa1215. PMID: 33417672

[114] Sfeir MM Meece JK Theel ES Granger D Fritsche TR Steere AC . Multicenter clinical evaluation of modified two-tiered testing algorithms for lyme disease using zeus scientific commercial assays. J Clin Microbiol. (2022) 60:e0252821. doi: 10.1128/jcm.02528-21. PMID: 35418241 PMC9116174

[115] Khan F Allehebi Z Shabi Y Davis I LeBlanc J Lindsay R . Modified two-tiered testing enzyme immunoassay algorithm for serologic diagnosis of lyme disease. Open Forum Infect Dis. (2022) 9:ofac272. doi: 10.1093/ofid/ofac272. PMID: 35873285 PMC9297310

[116] Landry ML Hassan S Rottmann BG Pesak SJ Ordazzo M Skrzyniarz M . Performance of two modified two-tier algorithms for the serologic diagnosis of Lyme disease. J Clin Microbiol. (2024) 62:e0013924. doi: 10.1128/jcm.00139-24. PMID: 38597655 PMC11077974

[117] Li Y Chen Z Tong CH Van K Jones RS Bare LA . Real-world Lyme disease testing results using modified vs standard two-tier test protocols. PloS One. (2025) 20:e0327376. doi: 10.1371/journal.pone.0327376. PMID: 40587524 PMC12208425

[118] Branda JA Steere AC . Laboratory diagnosis of lyme borreliosis. Clin Microbiol Rev. (2021) 34. doi: 10.1128/cmr.00018-19. PMID: 33504503 PMC7849240

[119] Maxwell SP Brooks C Kim D McNeely CL Cho S Thomas KC . Improving surveillance of human tick-borne disease risks: spatial analysis using multimodal databases. JMIR Public Health Surveill. (2023) 9:e43790. doi: 10.2196/43790. PMID: 37610812 PMC10483298

[120] Pretsch PK Tyrlik-Olk K Sandborn H Giandomenico DA Barbarin AM Williams C . Rapid Increase in Seroprevalence of Borrelia burgdorferi Antibodies among Dogs, Northwestern North Carolina, USA, 2017-2021(1). Emerg Infect Dis. (2024) 30:2047–55. doi: 10.3201/eid3010.240526. PMID: 39320158 PMC11431894

[121] Hahm JB Breneman J Liu J Rabkina S Zheng W Zhou S . A fully automated multiplex assay for diagnosis of lyme disease with high specificity and improved early sensitivity. J Clin Microbiol. (2020) 58. doi: 10.1128/jcm.01785-19. PMID: 32132190 PMC7180237

[122] Wang T Wang A Zindrili R Melis E Guntupalli S Brittain-Long R . Evaluation of the Epitogen Lyme Detect IgG ELISA: a novel peptide multiplexing approach. Microbiol Spectr. (2024) 12:e0167524. doi: 10.1128/spectrum.01675-24. PMID: 39436129 PMC11619319

[123] Levin AE Wormser GP Horn EJ Karaseva N Miller D Kellogg H . A novel single-tier serologic test to diagnose all stages of Lyme disease. J Clin Microbiol. (2025) 63:e0048325. doi: 10.1128/jcm.00483-25. PMID: 40833084 PMC12421848

[124] Li H Bai R Zhao Z Tao L Ma M Ji Z . Application of droplet digital PCR to detect the pathogens of infectious diseases. Biosci Rep. (2018) 38. doi: 10.1042/bsr20181170. PMID: 30341241 PMC6240714

[125] Leth TA Joensen SM Bek-Thomsen M Møller JK . Establishment of a digital PCR method for detection of Borrelia burgdorferi sensu lato complex DNA in cerebrospinal fluid. Sci Rep. (2022) 12:19991. doi: 10.1038/s41598-022-24041-8. PMID: 36411296 PMC9678864

[126] Molins CR Ashton LV Wormser GP Hess AM Delorey MJ Mahapatra S . Development of a metabolic biosignature for detection of early Lyme disease. Clin Infect Dis. (2015) 60:1767–75. doi: 10.1093/cid/civ185. PMID: 25761869 PMC4810808

[127] Pegalajar-Jurado A Fitzgerald BL Islam MN Belisle JT Wormser GP Waller KS . Identification of urine metabolites as biomarkers of early lyme disease. Sci Rep. (2018) 8:12204. doi: 10.1038/s41598-018-29713-y. PMID: 30111850 PMC6093930

[128] Zhou Y Qin S Sun M Tang L Yan X Kim TK . Measurement of organ-specific and acute-phase blood protein levels in early lyme disease. J Proteome Res. (2020) 19:346–59. doi: 10.1021/acs.jproteome.9b00569. PMID: 31618575 PMC7981273

[129] Henningsson AJ Lager M Brännström R Tjernberg I Skogman BH . The chemokine CXCL13 in cerebrospinal fluid in children with Lyme neuroborreliosis. Eur J Clin Microbiol Infect Dis. (2018) 37:1983–91. doi: 10.1007/s10096-018-3334-3. PMID: 30083887 PMC6154094

[130] Jung L Lâm TT Streng A Fingerle V Liese J . Elevated cerebrospinal fluid CXCL13 is a helpful marker for the early diagnosis of neuroborreliosis in children. Pediatr Infect Dis J. (2025) 44:577–81. doi: 10.1097/inf.0000000000004710. PMID: 39898650 PMC12058363

[131] Badawi A . The potential of omics technologies in lyme disease biomarker discovery and early detection. Infect Dis Ther. (2017) 6:85–102. doi: 10.1007/s40121-016-0138-6. PMID: 27900646 PMC5336413

[132] Bockenstedt LK Belperron AA . Insights from omics in lyme disease. J Infect Dis. (2024) 230:S18–26. doi: 10.1093/infdis/jiae250. PMID: 39140719 PMC12102470

[133] Haglund S Gyllemark P Forsberg P Brudin L Tjernberg I Henningsson AJ . Cerebrospinal fluid protein profiling of inflammatory and neurobiological markers in Lyme neuroborreliosis. Sci Rep. (2025) 15:20190. doi: 10.1038/s41598-025-06146-y. PMID: 40542080 PMC12181437

[134] Nielsen AB Fjordside L Drici L Ottenheijm ME Rasmussen C Henningsson AJ . The diagnostic potential of proteomics and machine learning in Lyme neuroborreliosis. Nat Commun. (2025) 16:9322. doi: 10.1038/s41467-025-64903-z. PMID: 41145505 PMC12559183

[135] Nayak S Sridhara A Melo R Richer L Chee NH Kim J . Microfluidics-based point-of-care test for serodiagnosis of Lyme Disease. Sci Rep. (2016) 6:35069. doi: 10.1038/srep35069. PMID: 27725740 PMC5057150

[136] Kim S Samanta K Nguyen BT Mata-Robles S Richer L Yoon JY . A portable immunosensor provides sensitive and rapid detection of Borrelia burgdorferi antigen in spiked blood. Sci Rep. (2023) 13:7546. doi: 10.1038/s41598-023-34108-9. PMID: 37161039 PMC10170079

[137] Ghosh R Joung HA Goncharov A Palanisamy B Ngo K Pejcinovic K . Rapid single-tier serodiagnosis of Lyme disease. Nat Commun. (2024) 15:7124. doi: 10.1038/s41467-024-51067-5. PMID: 39164226 PMC11336255

[138] Wagner L Obersriebnig M Hochreiter R Kadlecek V Larcher-Senn J Hegele L . Immunogenicity and safety of an 18-month booster dose of the VLA15 Lyme borreliosis vaccine candidate after primary immunisation in children, adolescents, and adults in the USA: a randomised, observer-blind, placebo-controlled, phase 2 trial. Lancet Infect Dis. (2026) 26:314–328. doi: 10.1016/S1473-3099(25)00541-9. PMID: 41213278

[139] Vannier E Richer LM Dinh DM Brisson D Ostfeld RS Gomes-Solecki M . Deployment of a Reservoir-Targeted Vaccine Against Borrelia burgdorferi Reduces the Prevalence of Babesia microti Coinfection in Ixodes scapularis Ticks. J Infect Dis. (2023) 227:1127–31. doi: 10.1093/infdis/jiac462. PMID: 36416014 PMC10175066

[140] Pine M Arora G Hart TM Bettini E Gaudette BT Muramatsu H . Development of an mRNA-lipid nanoparticle vaccine against Lyme disease. Mol Ther. (2023) 31:2702–14. doi: 10.1016/j.ymthe.2023.07.022. PMID: 37533256 PMC10492027

[141] Pfeifle A Lansdell C Zhang W Tamming LA Anderson-Duvall R Thulasi Raman SN . Polyvalent mRNA vaccine targeting outer surface protein C affords multi-strain protection against Lyme disease. NPJ Vaccines. (2025) 11:4. doi: 10.1038/s41541-025-01326-3. PMID: 41345132 PMC12774967

[142] Singh P Verma D Backstedt BT Kaur S Kumar M Smith AA . Borrelia burgdorferi BBI39 paralogs, targets of protective immunity, reduce pathogen persistence either in hosts or in the vector. J Infect Dis. (2017) 215:1000–9. doi: 10.1093/infdis/jix036. PMID: 28453837 PMC5407057

[143] Rios S Bhattachan B Vavilikolanu K Kitsou C Pal U Schnell MJ . The Development of a Rabies Virus-Vectored Vaccine against Borrelia burgdorferi, Targeting BBI39. Vaccines (Basel). (2024) 12. doi: 10.3390/vaccines12010078. PMID: 38250891 PMC10820992

[144] Gingerich MC Nair N Azevedo JF Samanta K Kundu S He B . Intranasal vaccine for Lyme disease provides protection against tick transmitted Borrelia burgdorferi beyond one year. NPJ Vaccines. (2024) 9:33. doi: 10.1038/s41541-023-00802-y. PMID: 38360853 PMC10869809

[145] Rios S Bhattachan B Wirblich C Chandwani A Vavilikolanu K Myers JF . Rabies virus-vectored Lyme disease vaccine provides long-term protection against tick-transmitted Borrelia burgdorferi. NPJ Vaccines. (2025) 10:231. doi: 10.1038/s41541-025-01294-8. PMID: 41233361 PMC12615636

[146] Sajid A Matias J Arora G Kurokawa C DePonte K Tang X . mRNA vaccination induces tick resistance and prevents transmission of the Lyme disease agent. Sci Transl Med. (2021) 13:eabj9827. doi: 10.1126/scitranslmed.abj9827. PMID: 34788080

[147] Vallery-Radot R . The life of Pasteur. In: Translation from the French by Mrs RL Devonshire, vol. II. London Constable (1902).

